# Detection of Phospho-Sites Generated by Protein Kinase CK2 in CFTR: Mechanistic Aspects of Thr1471 Phosphorylation

**DOI:** 10.1371/journal.pone.0074232

**Published:** 2013-09-18

**Authors:** Andrea Venerando, Cinzia Franchin, Natasha Cant, Giorgio Cozza, Mario A. Pagano, Kendra Tosoni, Ateeq Al-Zahrani, Giorgio Arrigoni, Robert C. Ford, Anil Mehta, Lorenzo A. Pinna

**Affiliations:** 1 Department of Biomedical Sciences, University of Padova, and CNR Institute of Neurosciences, Padova, Italy; 2 Proteomic Center of Padova University, VIMM, and Padova University Hospital, Padova, Italy; 3 Faculty of Life Sciences, University of Manchester, Manchester, United Kingdom; 4 Department of Molecular Medicine, University of Padova, Padova, Italy; 5 Division of Cardiovascular and Diabetes Medicine, Ninewells Hospital and Medical School, University of Dundee, Dundee, United Kingdom; Cedars-Sinai Medical Center, United States of America

## Abstract

By mass spectrometry analysis of mouse Cystic Fibrosis Transmembrane-conductance Regulator (mCFTR) expressed in yeast we have detected 21 phosphopeptides accounting for 22 potential phospho-residues, 12 of which could be unambiguously assigned. Most are conserved in human CFTR (hCFTR) and the majority cluster in the Regulatory Domain, lying within consensus sequences for PKA, as identified in previous mammalian studies. This validates our yeast expression model. A number of phospho-residues were novel and human conserved, notably mouse Ser670, Ser723, Ser737, and Thr1467, that all lie in acidic sequences, compatible with their phosphorylation by protein kinase CK2. Thr1467 is localized in the C-terminal tail, embedded in a functionally important and very acidic sequence (EETEEE) which displays an optimal consensus for protein kinase CK2. Herein, we show that Thr1467, homologous to human Thr1471 is readily phosphorylated by CK2. Indeed a 42 amino acid peptide encompassing the C-terminal segment of human CFTR is readily phosphorylated at Thr1471 with favorable kinetics (Km 1.7 µM) by CK2 holoenzyme, but neither by its isolated catalytic subunit nor by other acidophilic Ser/Thr kinases (CK1, PLK2/3, GCK/FAM20C). Our finding that by treating CFTR expressing BHK cells with the very specific CK2 inhibitor CX4945, newly synthesized wild type CFTR (and even more its Phe508del mutant) accumulates more abundantly than in the absence of CK2 inhibitor, supports the conclusion that phosphorylation of CFTR by CK2 correlates with decreased stability of the protein.

## Introduction

Cystic fibrosis (CF) is caused by mutations affecting a polytopic integral membrane protein, termed Cystic Fibrosis Transmembrane-conductance Regulator (CFTR) functioning as a phosphorylation stimulated, ATP-dependent anion channel. Pathogenic mutations of CFTR either impair its anion channel functions or make the protein unstable determining its premature degradation. This latter event is promoted by the commonest mutation of CFTR, a deletion of the amino acid residue phenylalanine 508 (Phe508delCFTR), which is found in 70–90% of CF patients. Phe508delCFTR is only partially functional when it is induced to traffic to the cell membrane [1], [2], but >99% of the protein undergoes premature degradation being targeted to the proteasome by the ER quality control machinery before it reaches the membrane [3]. Even the minor aliquot of Phe508delCFTR that reaches the membrane, moreover, displays a half-life much shorter than that of the wild type CFTR [4], further highlighting the intrinsic instability of this mutant.

In an attempt to disclose molecular events that may underlie deregulation of CFTR functionality and/or stability, attention has been focused on post-translational events, with special reference to phosphorylation. These studies clearly demonstrated that multiple phosphorylation of the regulatory domain of CFTR by PKA and probably also by other basophilic protein kinases such as PKC is essential to confer the full channel functionality to CFTR. The same residues phosphorylated in vitro by PKA [5] (and in some cases by PKC) were also found to undergo phosphorylation in living cells [6], [7], thus providing the clear-cut demonstration of the physiological occurrence of these events. It was also found in mutation studies that no single PKA site was dominant towards CFTR function and even a mutant with all PKA sites deleted retained some CFTR function.

Intriguingly however CFTR also includes several potential phospho-acceptor sites that display the consensus for the most pleiotropic acidophilic protein kinase, CK2 (an acronym derived from the old misnomer “casein kinase 2” [8]), but none of these has been ever reported to be phosphorylated in vivo, although the phosphorylation of a couple of these could be demonstrated in vitro [9]. While the PKA sites, whose phosphorylation has been validated in vivo are concentrated in the R domain (where they play a prominent role in the activation of the channel function) the potential CK2 sites are also localized outside the R domain and, by analogy with other CK2 targets whose phosphorylation commits them to degradation (see e.g. [10]–[12]), they may be implicated in the premature proteolysis of CFTR, also consistent with the outcome of recent mutational studies [13], [14]. It should be noted in fact that, although wild type CFTR is much more stable than its Phe508del counterpart, it nevertheless undergoes a very stringent quality control as well, resulting in perhaps 50% of the protein being discarded and proteolytically degraded [15]. Such a process is exceedingly complicated involving a network of proteins which includes chaperones [16], [17], glycan processing enzymes as well as the basal trafficking machinery [18], [19]. In this context CK2 may play a critical role in promoting accelerated degradation of CFTR, thus accounting for the failure to detect the phospho-form of CK2 phosphoacceptor sites in full length CFTR from living cells.

We reasoned that to circumvent this problem, advantage could be taken of CFTR expressed in yeast, where CK2 (as well as PKA and many other Ser/Thr protein kinases) is abundantly expressed and where a methodology for the expression of CFTR by a protocol that overcomes proteolysis problems has been recently set up [20]. By this method the degradation of CFTR is minimized providing good yields of pure CFTR exploitable for structural studies, but also useful for evaluation of its phosphorylation by endogenous Ser/Thr kinases minimizing complications arising from premature degradation.

By MS analysis of CFTR expressed in yeast we have now been able to show that besides all the “canonical” PKA sites concentrated in the R domain (whose detection demonstrates the reliability of the “yeast” approach) we additionally observe that some novel phospho-residues previously never found in CFTR from animal cells, are present in mouse CFTR expressed in yeast. One of these is the best predicted site for CK2, Thr1467 that is conserved in human CFTR (Thr1471): this residue is also readily phosphorylated in vitro with favourable kinetics by CK2 holoenzyme once included in a recombinant fragment of human CFTR encompassing its C-terminal segment. This outcome is described in the present report together with pharmacological evidence supporting the view that CFTR phosphorylation by CK2 may correlate with accelerated degradation of the protein.

## Materials and Methods

### Materials

All chemicals, cell culture media, anti-β tubulin antibody, and phosphatase inhibitor cocktails 2 and 3 were from Sigma-Aldrich. [γ-^33^P]ATP (3000 Ci/mmol) as well as HRP (horseradish peroxidase)-conjugated antibodies were purchased from PerkinElmer. Mouse monoclonal anti-CFTR antibody (10B6.2) that recognizes a specific epitope on NBD1 domain was from CFTR folding consortium (www. cftrfolding.org). Complete protease inhibitor cocktail tablets were from Roche Diagnostic. Okadaic acid was purchased from Alexis Biochemicals. Methotrexate was from Santa Cruz Biotechnology. CX4945 was from SYN|thesis med chem.

### Cloning, Expression and Purification of CFTR

Human CK2, both the α-catalytic subunit and the α_2_β_2_-holoenzyme were expressed in *Escherichia coli*. CK2α_2_β_2_ holoenzyme was reconstituted by mixing pellets expressing CK2α and CK2β independently. Cells were lysed by French press at 4°C in Tris/HCl 25 mM, pH 8, NaCl 0.4 M, DTT 1 mM, proteases inhibitors cocktail (Roche). The soluble fraction obtained after 30 min centrifugation at 15000 g was applied on Heparin-Sepharose and the proteins were separated using a gradient elution (0.4–1 M NaCl). Fractions containing CK2 were further purified by size-exclusion chromatography using a buffer composed by Tris/HCl 25 mM, pH 8, NaCl 0.5 M, DTT 1 mM.

CK1 isoforms and PLK 2 and 3 were obtained as described in [21], and [22], respectively.

Recombinant mouse full length CFTR (mCFTR) was cloned, expressed and purified as described in [20]. A 42-mer peptide reproducing the C-terminal tail of human CFTR (residues 1439–1480) was expressed in *Escherichia coli* with an N-terminal His tag and purified using affinity chromatography (Talon) followed by cleavage of the His tag by thrombin. Further details are given in the Material S1.

Synthetic peptide encompassing the last 20 residues of human CFTR C-terminal domain (KPQIAALKEETEEEVQDTRL, residues 1461–1480) was obtained by solid phase peptide synthesis method using a multiple peptide synthesizer (SyroII, MultiSynTech GmbH) on p-Benzyloxybenzyl alcohol resin (100–200 mesh) HMP resin (Novabiochem). The fluoren-9-ylmethoxycarbonyl (Fmoc) strategy was used throughout the peptide chain assembly, utilizing O-(7-Azabenzotriazol-1-yl)-N,N,N′,N′-tetramethyluronium hexafluorophosphate (HATU) as coupling reagent [23]. The side-chain protected amino acid building blocks used were: N-α-Fmoc-Nω-(2,2,4,6,7-pentamethyldihydrobenzofuran-5-sulfonyl)-L-arginine, N-α-Fmoc-γ-tert-butyl-L-glutamic acid, N-α-Fmoc-β-tert-butyl-L-aspartic acid, N-α-Fmoc-O-tert-butyl-L-threonine, N-α-Fmoc-Nε-(tert-butyloxycarbonyl)-L-lysine, N-α-Fmoc-N-γ-trityl-L-glutamine. Cleavage of the peptide was performed by reacting the peptidyl-resin with a mixture containing TFA/ethanedithiol/phenol 5% for 2.5 h. Crude peptide were purified by a preparative reverse phase HPLC. Molecular mass of the peptide were confirmed by mass spectroscopy on a MALDI TOF-TOF mass spectrometer (model 4800– AB Sciex). The purity (>90%) was evaluated by analytical reverse phase HPLC.

### MS Analysis

#### Trypsin digestion

Murine CFTR and the C-terminal peptide from human CFTR were digested in-gel with trypsin. Briefly, gel band was cut in small pieces of about 1 square mm and washed with several changes of 50% acetonitrile (ACN)/25 mM NH_4_HCO_3_. Gel pieces were then dried under vacuum and rehydrated with 50–100 µL of freshly prepared 10 mM DTT in 25 mM NH_4_HCO_3_. Reduction was carried on for 1 h at 56°C. DTT solution was discarded and a freshly prepared solution of 55 mM iodoacetamide in 25 mM NH_4_HCO_3_ was added to the gel pieces to alkylate cysteine residues for 45 min at room temperature in the dark. Gel pieces were then washed 3 times (10 min every time) alternating 25 mM NH_4_HCO_3_ and 50% ACN. The last washing step was done with 100% ACN and gel pieces were dried under vacuum. 20 µL of a solution 12.5 ng/µL in 25 mM NH_4_HCO_3_ of sequencing grade trypsin (Promega) was added to the gel sample and rehydration was carried on for 45 min at 4°C. Digestion was then performed overnight at 37°C. Peptides were extracted with 3 changes of 50% ACN/0.1% formic acid (30 min between changes). Sample was dried under vacuum kept at −20°C until MS analyses were performed.

#### Phosphopeptides enrichment

Phosphopeptides derived from the trypsin digestion of CFTR were enriched with micro-columns of TiO_2_ prepared in house as described in [24]. Sample was dissolved in 20 µL of loading buffer consisting of 80% ACN/6% trifluoroacetic acid (TFA). The micro-column was conditioned twice (20 µL each time) with ACN and twice with loading buffer. Sample was loaded and slowly pushed through the column, which was then washed twice (20 µL each) with loading buffer and twice with a washing buffer (0.1% TFA). Phosphopeptides bound to TiO2 were eluted with 20 µL of freshly prepared 5% NH_4_OH and subsequently with 20 µL of 50% ACN/0.1% formic acid. Sample was immediately acidified by adding 2 µL of 100% formic acid and dried under vacuum.

#### LC-MS/MS and data analysis

Mass spectrometry analysis of phosphopeptides was performed with a LTQ-Orbitrap XL hybrid mass spectrometer (Thermo Fisher Scientific) coupled online with a nano-HPLC Ultimate 3000 (Dionex-Thermo Fisher Scientific). Sample was dissolved in 20 µL of 0.1% formic acid (FA) and for every analysis 5 µL of sample were loaded at a flow rate of 8 µl/min into a trap column (300 µm I.D., 300 Å, C18, 3 µm; SGE Analytical Science). Sample was then injected with a flow rate of 250 nL/min into a 10 cm pico-frit capillary column (75 µm I.D., 10 µm tip; New Objectives) home-packed with C18 resin (Reprosil 300Å, 3 µm). Peptides were separated using a linear gradient of ACN/0.1% FA from 3% to 40% in 20 min.

MS analyses were performed with 3 different acquisition methods, using the same chromatographic conditions. A MS2 data dependent acquisition (1 MS scan on the Orbitrap with a resolution of 60000, followed by MS/MS spectra acquired in the linear ion trap for the 10 most abundant peptides); a MS3 neutral loss-triggered dependent acquisition (1 MS scan on the Orbitrap with a resolution of 60000, followed by MS/MS scans on the 3 most intense ions and by MS3 upon detection of neutral loss of phosphoric acid in MS2 spectra); a Multi Stage Acquisition (MSA) (1 MS scan at a resolution of 60000 followed by MS/MS scans on the 3 most abundant ions with the activation of neutral loss product without an additional isolation cycle).

Raw data files were analyzed with the Proteome Discoverer software version 1.3 (Thermo Fisher Scientific) connected to a Mascot Server version 2.2.4 (Matrix Science, UK) and a Sequest search engine version 28.0 (Thermo Fisher Scientific) against the Uniprot/SwissProt Database (version 20110526). Trypsin was set as digesting enzyme with up to 1 missed-cleavage. Cabamidomethyl cysteine was set as fixed modification, while phosphorylation of Ser/Thr/Tyr and methionine oxidation were set as variable modifications. Peptide and fragment tolerance were 5 ppm and 0.6 Da respectively. False Discovery Rate (FDR) based on the search against the corresponding randomized database and Percolator were used to discriminate between correct and decoy spectrum identifications. The software phosphoRS [25] was used to help in the correct assignment of phosphorylation sites.

Files were analyzed using both Mascot and Sequest and searched for MS2 and MS3 spectra; all results were combined directly by Proteome Discoverer into a single multi-report file. Spectra of phosphorylated peptides were manually inspected for further confirmation of the results.

### Cell Culture

Baby hamster kidney cells (BHK) stably expressing human CFTR (hCFTR) either wild type (WT), or Phe508del, or WT T1471A were growth to reach 70–80% confluency [13]. After 2 h-long treatment with 100 µg/mL cyclohexamide, cells were washed with PBS and incubated with 2 µM CX4945 or its vehicle for the indicated time. At each time-point cells were washed with ice-cold PBS, scraped from the plates, pelleted by centrifugation (1200 g for 5 min at room tempertaure) and lysed in a buffer containing 1% (v/v) Nonidet P40, 50 mM Tris/HCl (pH 7.5) and 150 mM NaCl with fresh protease inhibitor tablets (Roche), 1 µM Okadaic acid (Alexis) and phosphatases inhibitors cocktails 2 and 3 (Sigma). Cells were lysed by incubation on ice for 30 min and then the lysates were cleared by centrifugation at 17000 g for 30 min at 4°C. The supernatant was collected and subjected to protein concentration quantification.

Western blot analysis was performed as described in [14] using a modified Laemmli buffer containing 105 mM dithiothreitol (final concentration) to enhance the solubilization of CFTR. 20–30 µg of total proteins were separated on 4–12% precast NuPage® Bis-Tris gradient gels (Invitrogen) according to the manufacturer’s instructions and transferred on to Optitran BA-S 83 nitrocellulose membranes (Whatman) with a semi-dry blotter (Biometra) for 1 h at 150 mA. Membranes were saturated for 1 h with 5% skimmed milk in Tris-buffered saline (TBS) containing 0.2% Tween 20. Immunoblots were developed by enhanced chemiluminescence, detected on Kodak Image Station 440 cf and analyzed by the Kodak 1D image software. Tubulin was used as loading control.

### Phosphorylation Assay

Peptides derived from human CFTR C-terminal region were phosphorylated by incubation in a 20-µl volume containing 50 mM Tris/HCl, pH 7.5, 10 mM MgCl_2_, 100 mM NaCl, and 50 µM [γ-^33^P]ATP (specific radioactivity 1,500–2,000 cpm/pmol). The reaction was started by the addition of the indicated protein kinases. The reaction mixtures were incubated for 10 minutes at 37°C and stopped by the addition of 2× concentrated Tricine sample buffer (450 mM Tris HCl, pH 8.45, 12% (v/v) glycerol, 4% (w/v) SDS, 0.0025% (w/v) Coomassie Blue G, 0.0025% (w/v) Phenol Red and 105 mM DTT). Samples were separated on Tricine-SDS-PAGE following the Schägger’s protocol [26] in a running buffer composed of 100 mM Tris/HCl, pH 8.3, 100 mM Tricine, 0.1% (w/v) SDS. ^33^P incorporation was evaluated by gels autoradiography on PerkinElmer’s Cyclone Plus Storage Phosphor System. Initial rate data were analyzed with the program Prism (GraphPad Software, La Jolla, CA).

### In Silico Analysis

Structural model of the last 42 aminoacids of CFTR was built using ab initio Rosetta tool [27] and generating 50000 models of the target sequence. These models were clusterized using the cluster application in order to identify the most frequently sampled conformations. The final model was submitted to a 100 ns Molecular Dynamics (MD).

Protein-protein docking analysis was performed using two FFT-based docking software PIPER [28] and Zdock [29]. The procedures were performed using the C-terminal model of CFTR as the probe and CK2 tetrameric structure (PDB code: 4DGL) as the target protein. 1000 complexes were obtained from both docking algorithms and clusterized using the pairwise RMSD (Root Mean Square Deviation) into 5 largest clusters. The final complex was chosen according to the energy scoring function.

Molecular dynamics (MD) simulations of the final complex (parameterized with AMBER99) were performed with NAMD 2.8 [30] in order to verify their stability over time; in particular a 100 ns of NPT (1 atm, 300 K) MD simulation were performed after an equilibration phase of 1 ns (positional restraints were applied on carbon atoms to equilibrate the solvent around the protein).

## Results

**Figure 1:**
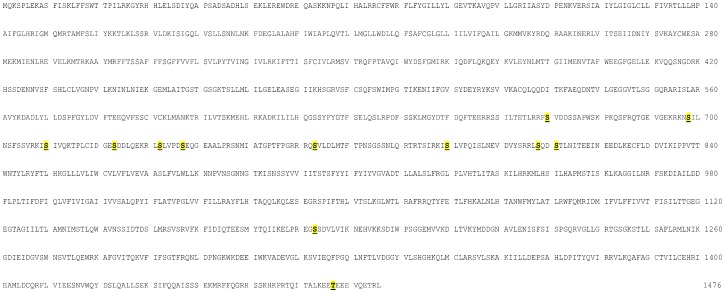
The sequence of mouse CFTR (accession number P26361) with all the unambiguously assigned phospho-sites identified.

Full length mouse CFTR (mCFTR) expressed in *Saccharomyces cerevisiae* was subjected to tryptic digestion followed by TiO_2_ phosphopeptide enrichment prior to MS analysis as detailed in the Experimental section. From the MS spectra (Material S1, Figures S1 and S2) it was possible to identify 21 phosphopeptides derived from CFTR (see Table S1) leading to the identification of 22 potential phospho-sites, 12 of which could be unambiguously assigned (see Table 1 and Figure 1). In a few cases it was not possible to assign the phosphate to individual Ser/Thr residue(s) within the phosphopeptides, thus hindering the precise localization of the phospho-residue(s). Almost all the phospho-sites identified in mouse CFTR are conserved in human CFTR (hCFTR), as also indicated in Table 1. The majority belong to the Regulatory (R) domain and display the consensus for PKA. Many of these residues were found to be phosphorylated in CFTR expressed in *Xenopus* oocytes [6] and/or in HEK293S cells [7]. The same residues were also phosphorylated in vitro by PKA [5], [6].

**Table 1:** Phospho-sites identified in mouse CFTR expressed in yeast.

phospho-residue number						
Mouse CFTR (P26361)	Human CFTR (P13569)	Domain	Sequence (mouse)	Phosphorylatedin vivo	Phosphorylated in vitro by PKA	Phosphorylated in vitro by CK2
S670	S670	R-domain	TETLRRF**S**VDDSSAP		[6]	[9]
S698	S700	R-domain	VGEKRKN**S**ILNSFSS	[6], [7]	[5], [6]	
S710	S712	R-domain	FSSVRKI**S**IVQKTPL	[6], [7]	[5], [6]	
S723	S728	R-domain	PLCIDGE**S**DDLQEKR			
S732	S737	R-domain	DLQEKRL**S**LVPDSEQ	[6], [7]	[5], [6]	
S737	S742	R-domain	RLSLVPD**S**EQGEAAL			
S763	S768	R-domain	FPGRRRQ**S**VLDLMTF	[6]	[5], [6]	
S790	S795	R-domain	RTSIRKI**S**LVPQISL	[6], [7]	[5], [6]	
S808	S813	R-domain	DVYSRRL**S**QDSTLNI		[5], [6]	
S811	T816	R-domain	SRRLSQD**S**TLNITEE			
S1183	–	–	KELPREG**S**SDVLVIKN			
T1467	T1471	C-term	ITALKEE**T**EEEVQET			This paper

Several phospho-residues identified by our analysis however were not previously reported to be phosphorylated in vivo: one or more are located in the N-terminal segment (Ser45 and/or Ser50) and at the end of the R domain (Ser808 and Ser811), most of which are conserved in hCFTR. Also conserved is Ser670 that lies at the end of the R domain, whose in vitro phosphorylation by both PKA [6] and CK2 [9] was reported but which hitherto has never been observed in vivo.

Phospho-Thr1467, close to the C-terminus and sitting in a region which is 100% conserved in hCFTR (Thr1471), attracted our attention for three reasons: i) it conforms to the optimal sequence recognized by CK2, ii) it is adjacent to the PDZ interacting motif which is essential for interaction with AMP-activated protein kinase [31] and with the PDZ domain of the scaffold protein NHERF-1 [32], [33] and iii) it is embedded in an acidic cluster that plays a role in regulating CFTR gating and appears to increase the amount of intracellular protein [34].

**Figure 2:**
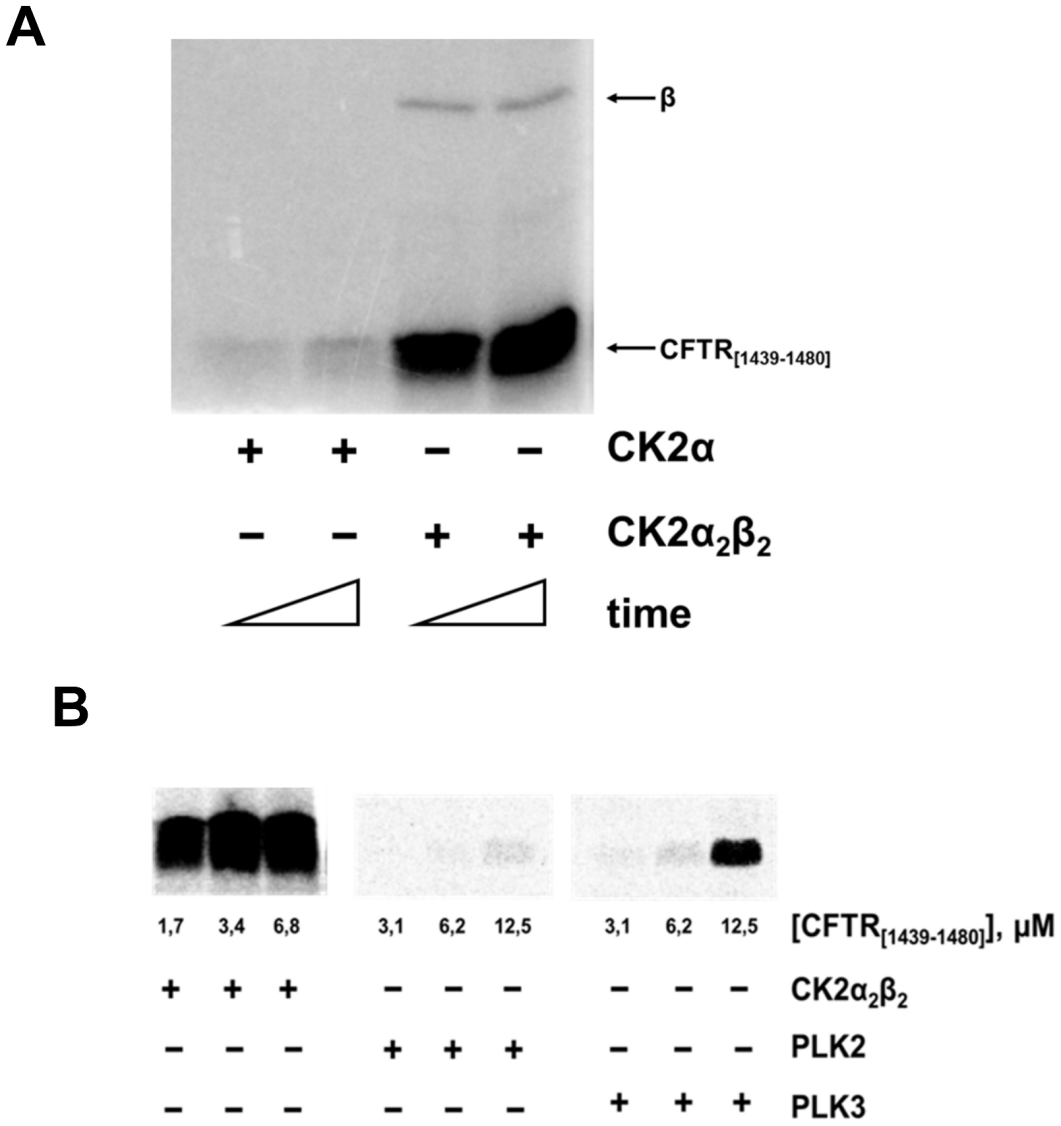
C-terminal domain of human CFTR (hCFTR) is readily phosphorylated by CK2 holoenzyme. (A) 0.4 µg of recombinant C-terminal tail of CFTR encompassing the last 42 residues of the human sequence was phosphorylated as described in Materials and Methods section in the presence of either CK2 α-catalytic subunit or CK2 holoenzyme (CK2 α_2_β_2_). The arrows indicate 42-mer CFTR peptide (CFTR_[1439–1480]_) phosphorylation and β-subunit autophosphorylation, respectively. (B) Phosphorylation of the 42-mer peptide in the same conditions as in (A) by PLK2 and PLK3, is very low as compared to that obtained with CK2 holoenzyme.

**Figure 3:**
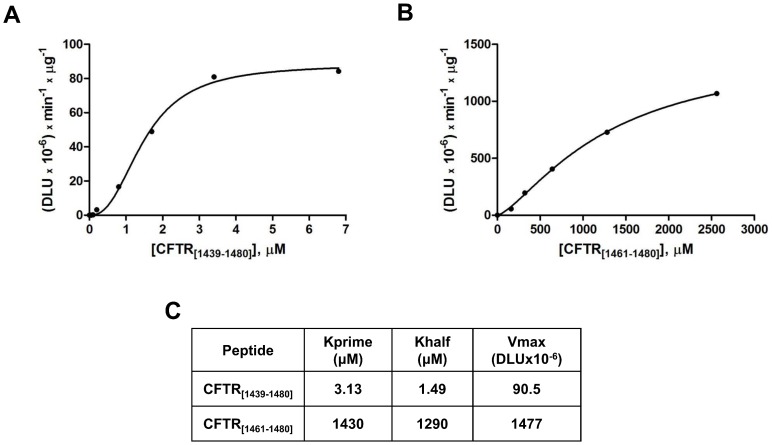
Kinetics of the recombinant 42-mer peptide (A) and of a synthetic peptide reproducing the last 20 residues of the hCFTR C-term (CFTR_[1461–1480]_) (B). Data obtained did not follow the Michaelis-Menten equation but fitted to an allosteric (sigmoidal) model. Kinetic parameters displayed in (C) were calculated by GraphPad Prism program. Kprime (also described as Khalf^h^, where h is the Hill slope) is related to Km but it is not equal to it unless h = 1.

**Figure 4:**
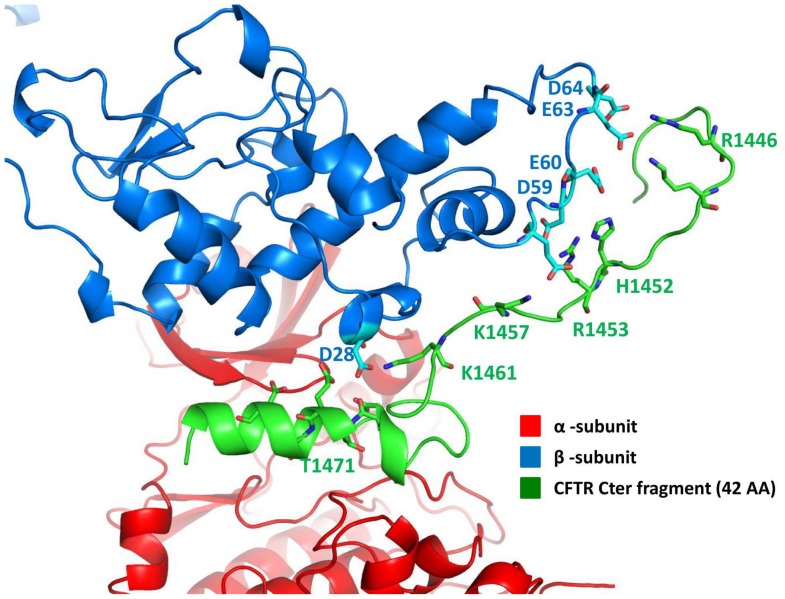
Protein-protein docking complex between the tetrameric structure of CK2 (PDB code: 4DGL) and the hCFTR peptide containing the last 42 amino acids.

To corroborate the implication of CK2 as a phosphorylating agent of CFTR Thr1471 a 42 amino acid-long recombinant fragment encompassing the C-terminal segment of hCFTR was generated and tested for its phosphorylation by CK2. As shown in Figure 2A the peptide is readily phosphorylated by CK2 holoenzyme, composed of two catalytic subunits assembled with a dimer of the regulatory β-subunits, but not by the isolated catalytic α-subunit alone, a behavior typical of class III substrates of CK2 [8] that require the structural β-subunit for recognition by the holoenzyme (see below). Phosphorylation occurs at Thr1471 as revealed by MS analysis of the phosphorylated 42 aa fragment (Material S1, Figure S3). Consistently a synthetic peptide encompassing the last 20 residues of hCFTR, in which the only phosphorylatable residue is Thr1471 can also be phosphorylated by CK2 holoenzyme, although much less efficiently (Figure 3). To note in this respect the quite low Km of the 42 amino acids fragment (1.4 µM) a value denoting high affinity and typically displayed by physiological targets rather than by short peptide substrates. These biochemical data are in very good agreement with an in silico study whose outcome is summarized in Figure 4. This predicts that the binding of the hCFTR C-terminal tail to CK2 holoenzyme is critically mediated by interactions between an acidic cluster present in the N-terminal region of the CK2 β-subunit (residues Glu57, Asp59, Glu60, Glu63, Asp64) and a number of basic side chains clustered in CFTR up-stream from Thr1471 (residues Arg1446, Lys1448, His1452, Arg1453, Lys1457). Of note, the latter are present in the recombinant fragment of 42 residues, but not in the shorter 20 amino acids peptide. It is hence quite expected that optimal phosphorylation of Thr1471 will require both the integrity of the CFTR C-terminal segment and the presence of the β-subunit, as highlighted by the experiments in Figure 2A.

We also determined whether other acidophilic Ser/Thr protein kinases, besides CK2, are able to phosphorylate Thr1471. While protein kinases CK1 and the genuine Golgi apparatus casein kinase (GCK), recently identified with FAM20C protein [35], [36] proved unable to phosphorylate the 42 amino acid peptide (not shown), a weak phosphorylation was observed using PLK2 and PLK3 recently shown to recognize residues specified by acidic determinants [37], [38]. Such a phosphorylation however is negligible as compared to that catalyzed by CK2 under comparable conditions (Figure 2B).

Given the location of Thr1471 close to the PDZ-interacting motif that favors retention of CFTR within the cell by interacting with Golgi associated CAL (Cheng J et al JBC 2002) [39] and its competitor MAST205 [40], we argued that its phosphorylation could affect the stability of such interactions and consequently the turnover of CFTR itself. Accordingly a hCFTR mutant no longer susceptible to such a phosphorylation (T1471A) was overexpressed in BHK cells as compared to hCFTR wild type. This mutant was previously shown to fragment in a manner similar to the wild type protein [14].

**Figure 5:**
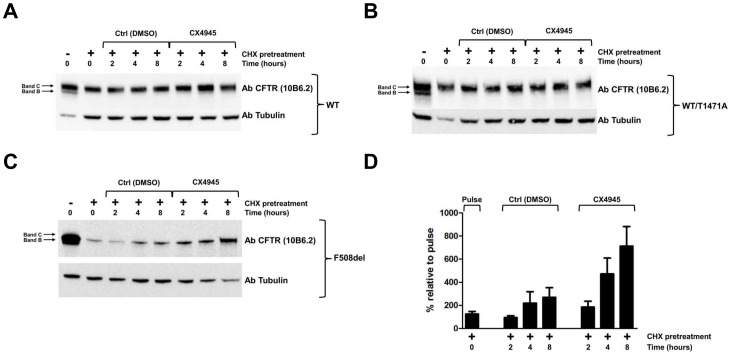
Effect of CK2 inhibition on Phe508delCFTR synthesis. BHK cells expressing human CFTR wild type (A), T1471A (B) and Phe508del (C) were treated for 2 h with CHX to stop protein synthesis (compare first and second lanes). Cells were washed with PBS and incubated for the indicated time with either CK2 specific inhibitor CX4945 or its vehicle. Proteins from each sample were separated by gel electrophoresis and blotted on nitrocellulose membranes (see Materials and Methods). Blots were developed for the indicated antibodies. In (D) quantitation of Phe508delCFTR band B expression after the indicated treatment is shown. The figure is representative of experiments performed in triplicate. Due to the low expression level, Phe508delCFTR signal was obtained by overexposed conditions.

To provide evidence about the implication of CK2 in processes affecting the stability of CFTR and/or of its Phe508del mutant which is the commonest cause of Cystic Fibrosis, advantage has been taken of the most selective cell permeable CK2 inhibitor available, CX4945. In order to evaluate the efficacy of CX4945 on the level of de novo synthesized CFTR, protein synthesis was inhibited by two hours cell treatment with cycloheximide (CHX). Blockade was then removed by washing off CHX and newly synthesized CFTR was quantified after 2, 4, and 8 hrs incubation either in the absence or presence of CX4945. As shown in Figure 5 treatment with CHX causes a fast and almost complete disappearance of band B, corresponding to immature core-glycosylated CFTR, whereas the decrease of band C, corresponding to mature fully glycosylated CFTR, where present (Panels A and B), is much slower, its degradation being partially compensated by the conversion of band B into C, a process which is not prevented by CHX. This point is further highlighted by comparing CHX and Brefeldin A, a compound that blocks the escape of core-glycosylated CFTR from Endoplasmic Reticulum to the Golgi, displaying opposite effects on the fate of band B as shown in Figure S4. To note that in the case of Phe508delCFTR (Figure 5, Panel C) band C is undetectable and therefore the disappearance of band B is more dramatic being not accompanied by any generation of band C. Both these behaviors were expectable, considering the inability of Phe508delCFTR to undergo maturation and its special proneness to premature degradation. Once blockade is removed de novo synthesis of CFTR wild type is significantly stimulated if endogenous CK2 is inhibited by cell treatment with CX4945 (Panel A), a similar effect of CX4945 being also detectable if wild type CFTR is replaced by its T1471A mutant, which is no longer susceptible to phosphorylation at Thr1471 (Panel B). By contrast the efficacy of inhibiting CK2 is enhanced if the de novo synthesis of Phe508delCFTR is monitored (Panel C). This point has been corroborated by performing the experiment in triplicate and drawing from it quantification of Phe508delCFTR band B, as reported in Panel D.

It can be concluded from the data of Figure 5 that inhibition of CK2 correlates with increased stability of CFTR, which is particularly evident in the case of Phe508delCFTR. Phosphorylation of Thr1471 seems not to be instrumental to such an effect, at least in a CFTR wild type background.

## Discussion

Expression of CFTR in yeast was primarily aimed at producing sufficient amount of the protein for structural studies [20]. We reasoned however that an added value of this method could be that of making possible the detection of phosphorylated residues which may be lost if the expression is performed in animal cells. We were impressed in particular by the observation that, although several potential phosphoacceptor sites for CK2 are present in CFTR and some of these are readily phosphorylated by CK2 in vitro [9] none of these have been ever reported to become phosphorylated in vivo, in sharp contrast with potential PKA phosphoacceptor sites for many of which a good correlation has been shown to exist between in vitro and in vivo phosphorylation [5]–[7]. Considering on the one hand the stringent quality control experienced by CFTR, leading to the destruction of almost half of the newly synthesized protein, and, on the other, the role frequently played by CK2 mediated phosphorylation to commit proteins to degradation (e.g. [10]–[12]) a possibility would be that in its physiological environment CFTR phosphorylation by CK2 cannot be detected because it is immediately followed by CFTR fragmentation.

Based on this supposition we wanted to see if CFTR expressed in a foreign environment like yeast, where CK2 (as well as PKA and many other important Ser/Thr protein kinases) are abundant, but where the homeostatic mechanism leading to CFTR degradation is lacking and proteolysis has been prevented by a number of devices, CFTR is phosphorylated, and at which residues, by CK2.

The study presented here led to the identification of 22 potential phospho-residues (12 of which were unambiguously assigned) in mouse CFTR expressed in yeast, all of which, with just a few exceptions, are conserved in human CFTR. Seven of these coincide with phospho-residues previously identified in human CFTR expressed in mammalian cells and/or *Xenopus* oocytes [6], [7], thus corroborating the reliability of the yeast expression approach. These phospho-residues, with only one exception are located within the Regulatory (R) domain of CFTR and they display the consensus for being phosphorylated by PKA. Accordingly these residues are also included in the list of those which are phosphorylated in vitro by PKA [5], [6].

To the best of our knowledge the other phospho-residues detected by us in CFTR were not reported before: one however (Ser670) was previously reported to undergo in vitro phosphorylation by both PKA [6] and CK2 [9] but was never reported as an in vivo site. It actually displays the consensus for both kinases (RR-x-S, and S-x-x-E/D): whether PKA or CK2 or maybe both are committed with its phosphorylation in vivo remains a matter of conjecture.

At variance with the previously identified CFTR phospho-sites, which are almost exclusively located in the R domain and display the PKA consensus, several of the newly identified ones described here are located close to acidic residues which may suggest their targeting by CK2 and some are located outside the R domain: in particular one or two are located in the N-terminal segment, but could not be assigned with good confidence to individual positions (see Material S1), though it is possible that serines 45 and 50 are both phosphorylated. None of these conforms to either the PKA or CK2 consensuses, while another phospho-site outside the R domain, around phospho-Thr1467 (100% conserved in human CFTR as Thr1471) displays an optimal sequence for being phosphorylated by CK2. Two other phospho-residues which, though not fulfilling the canonical CK2 consensus are close to multiple acidic side chains which could nevertheless determine their phosphorylation by CK2 [41] are Ser723, homologous to hCFTR Ser728 which is located inside a PEST region [42] where it could affect degradation, and Ser737 whose human equivalent (Ser742) is surrounded by an identical sequence and was never reported to be phosphorylated before.

Our attention was mainly focused on phospho-Thr1471 (human numbering), since besides being highly conserved in human CFTR and displaying an optimal sequence for CK2 phosphorylation, it is adjacent to the PDZ interacting motif which is essential for interactions with the scaffold protein NHERF-1, anchoring CFTR to the cytoskeleton [32], [33] and with the Golgi associated CAL favoring CFTR retention into the cell [39]. To validate the implication of CK2 in the phosphorylation of CFTR Thr1471 we have firstly shown that a recombinant fragment of human CFTR encompassing it is readily phosphorylated, with favorable kinetics, at Thr1471 by CK2 holoenzyme but not by a number of other acidophilic protein kinases. Secondly we demonstrated that a shorter synthetic peptide encompassing Thr1471 is also phosphorylated by CK2 albeit with lower efficiency, as expected from in silico analysis showing that optimal binding of the CFTR C-terminal tail to CK2 holoenzyme requires the integrity of a segment which is present in the recombinant fragment but not in the synthetic peptide. These interactions take place outside the catalytic subunit of CK2, involving elements of the β-subunit: this includes CFTR in the list of class III CK2 substrates [8] whose phosphorylation is readily catalyzed by the holoenzyme (composed by two catalytic and two β-subunits) but not by the isolated catalytic α-subunits.

To get clues about the possible consequences of CFTR phosphorylation by CK2, advantage has been taken of the most selective CK2 inhibitor available to date, CX-4945 [43] and of a CFTR mutant in which Thr1471 has been replaced by alanine (T1471A) [13].

By treating CFTR expressing BHK cells with cycloheximide, in order to block protein synthesis and then restoring protein synthesis by washing away the inhibitor we observed that the re-appearance of CFTR reached higher levels if cells were incubated with the CK2 inhibitor CX-4945, as compared to control. This indicates that inhibition of CK2 results in larger accumulation of newly synthesized CFTR, consistent with diminished degradation of the protein. Interestingly such an effect is definitely more pronounced in the case of Phe508delCFTR with a two to three-fold increment of band B upon treatment with the CK2 inhibitor (see Figure 5D), suggesting that CK2 specifically cooperates with the mechanism which leads to premature degradation of this mutant. On the other hand the effect of CK2 on CFTR stability seems not to be mediated by Thr1471, at least in a wild type background, since a similar effect of CX4945 was observed with CFTR wild type and its T1471A mutant (Figure 5, compare Panels A and B). It cannot be ruled out however that in a Phe508del background phosphorylation of Thr1471 may play a role in committing CFTR to premature degradation. Other possibilities worty of consideration are that the detrimental effect of CK2 on CFTR stability as outlined by the experiments shown in Figure 5 is promoted through the phosphorylation of residues other than Thr1471, with special reference to Ser728 (see Table 1), located near to a PEST region and/or indirectly, by altering the functionality of proteins committed to quality control, folding and degradation of CFTR [44], several of which have been shown to undergo phosphorylation by CK2 [45 and unpublished data].

Although additional work is needed in order to understand the significance of CFTR phosphorylation by CK2, with special reference to the enigmatic role of Thr1471, our data disclose the importance that a number of previously undetected phosphoacceptor sites may have in determining the stability and the functionality of CFTR. Our experiments also highlight the role of the master kinase CK2 in these processes, providing, from an empirical stand point, a new strategy to increase the yield of functional CFTR in CF cells, possibly by combining CK2 manipulation with usage of correctors and/or potentiators.

## Supporting Information

Figure S1 **The sequence of mouse CFTR with all phosphopeptides identified is reported together with peptide identification confidence and phosphorylation site probability that are indicated with a color code.** Table at the end of the file summarizes the data, by listing all identified phosphopeptides with their corresponding phosphorylation sites.
(TIF)

Figure S2 **Annotated fragmentation spectra of the phosphopeptides identified in this study.**
(PDF)

Figure S3 **MS/MS spectrum of phosphopeptide EETEEEVQDTR from Human CFTR.**
(TIF)

Figure S4 **BHK cells expressing WT, WT/T1471A or Phe508delCFTR were exposed to either cycloheximide (CHX, 100 µg/ml) or Brefeldin A (BFA, 200 ng/ml).** CFTR synthesis was blocked by two hours treatment with CHX as it is shown by the attenuation/disappearance of the ER-resident band B of CFTR. On the contrary the fully mature band C is not affected by CHX treatment due to its slower turnover. Conversely, when the escape from ER is prevented by using Brefeldin A, band B increases [a]. [a] Glozman R, Okiyoneda T, Mulvihill CM, Rini JM, Barriere H, Lukacs GL (2009) N-glycans are direct determinants of CFTR folding and stability in secretory and endocytic membrane traffic. J. Cell Biol. 184, 847–862.
(TIF)

Table S1 Phosphopeptides identified from mouse CFTR. For each peptide the following details are reported: aminoacid sequence, modifications, phosphorylation site/s, pRS score, pRS probability and pRS site probability, q-value, PEP score and number of missed-cleavages.
(DOC)

Material S1 (DOC)
