# Supplementary figures and images for: Detection of Phospho-Sites Generated by Protein Kinase CK2 in CFTR: Mechanistic Aspects of Thr1471 Phosphorylation
Source: PLoS One. 2013 Sep 18;8(9):e74232. doi: 10.1371/journal.pone.0074232 (PMC3776838; doi:10.1371/journal.pone.0074232)

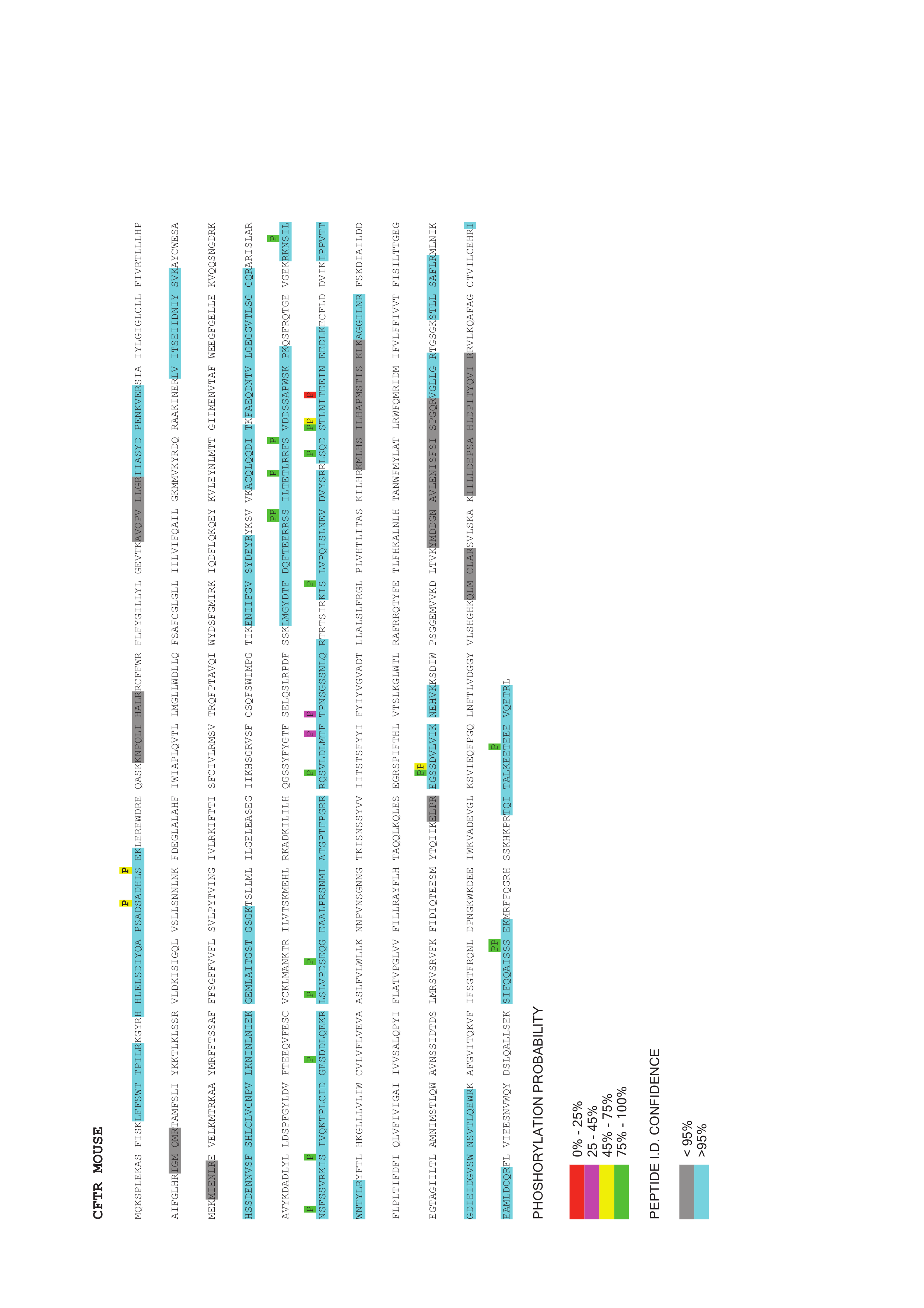

Supplement: Figure S1 — The sequence of mouse CFTR with all phosphopeptides identified is reported together with peptide identification confidence and phosphorylation site probability that are indicated with a color code. Table at the end of the file summarizes the data, by listing all identified phosphopeptides with their corresponding phosphorylation sites. (TIF) [file pone.0074232.s001.tif]

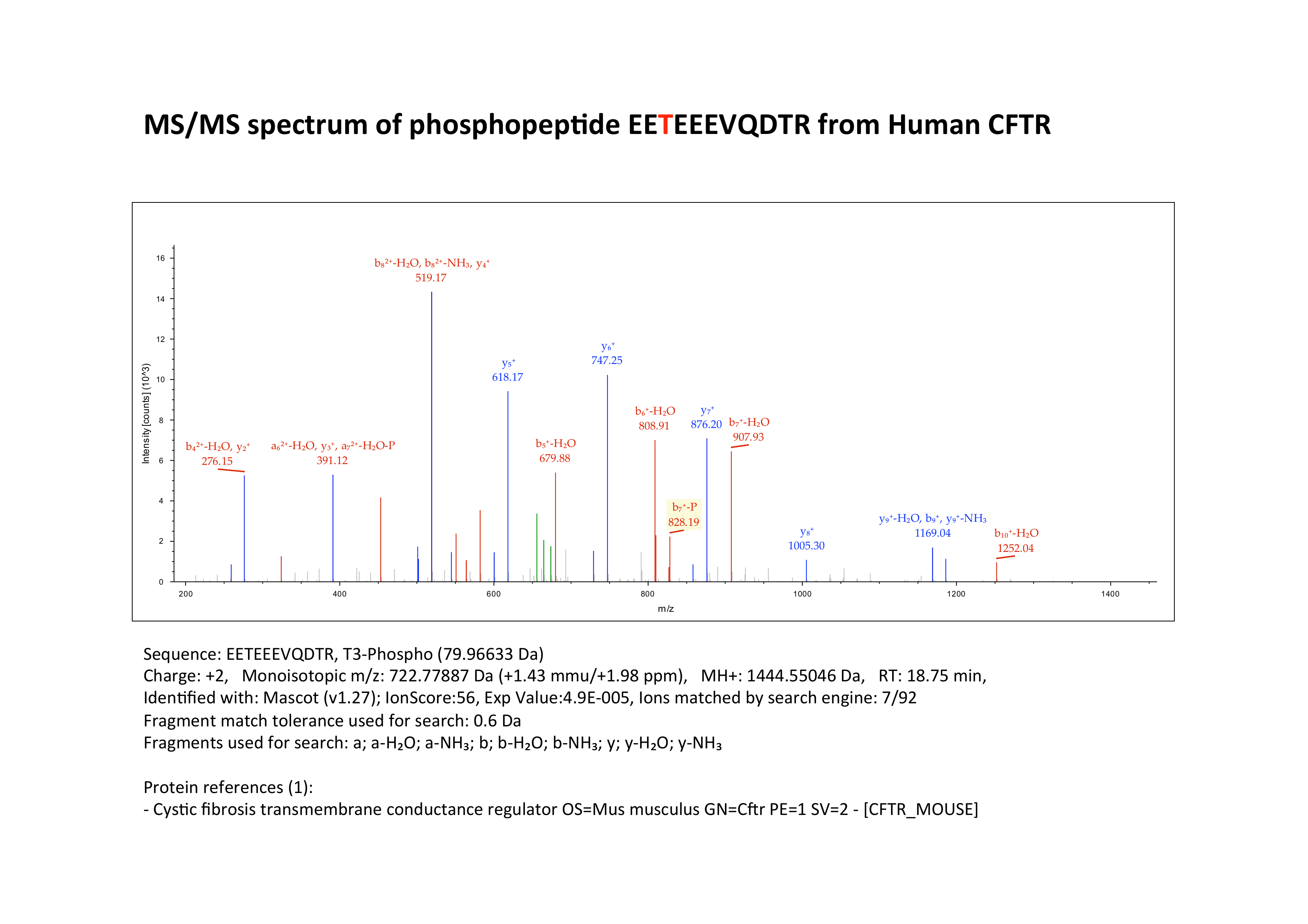

Supplement: Figure S3 — MS/MS spectrum of phosphopeptide EETEEEVQDTR from Human CFTR. (TIF) [file pone.0074232.s003.tif]

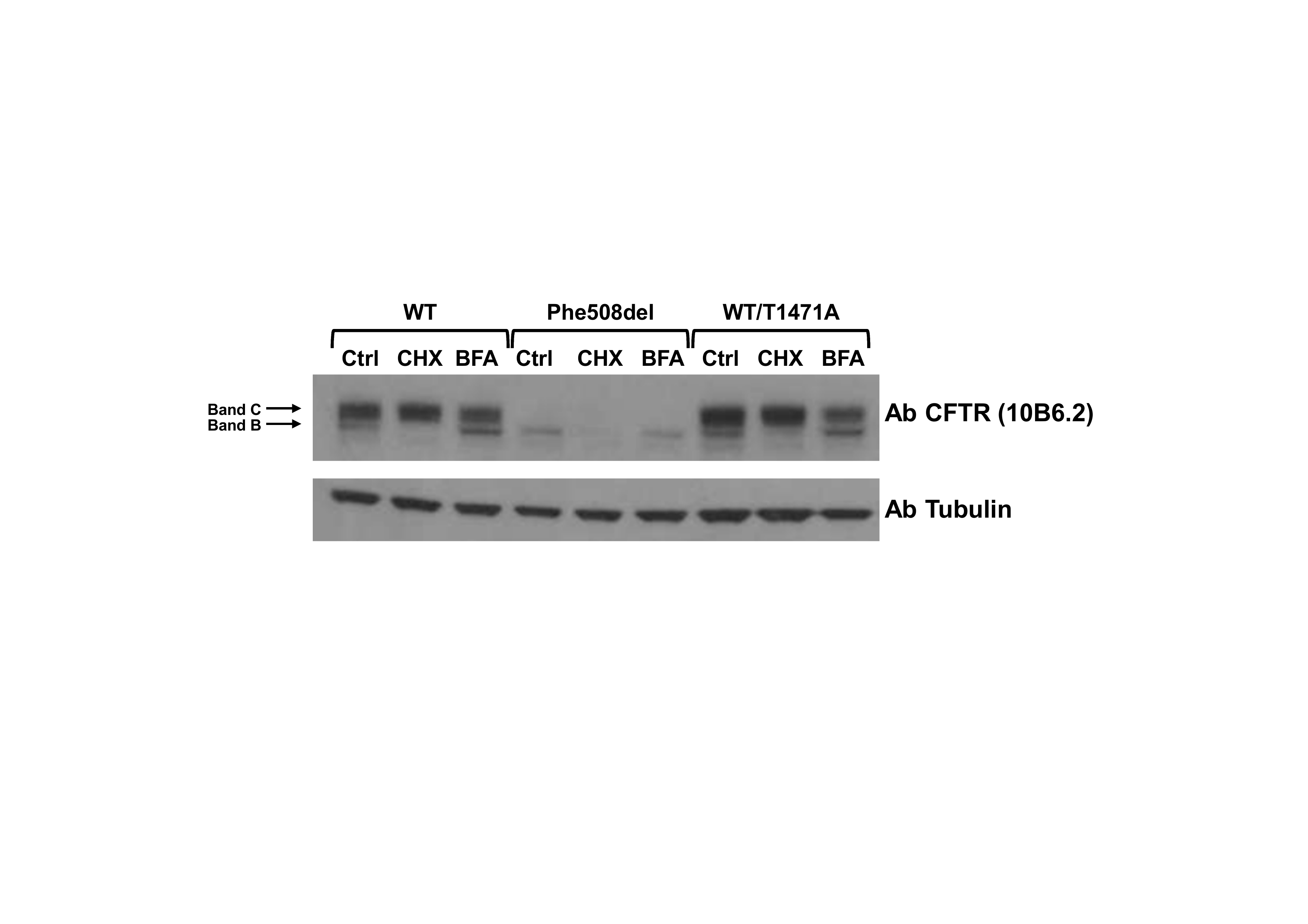

Supplement: Figure S4 — BHK cells expressing WT, WT/T1471A or Phe508delCFTR were exposed to either cycloheximide (CHX, 100 µg/ml) or Brefeldin A (BFA, 200 ng/ml). CFTR synthesis was blocked by two hours treatment with CHX as it is shown by the attenuation/disappearance of the ER-resident band B of CFTR. On the contrary the fully mature band C is not affected by CHX treatment due to its slower turnover. Conversely, when the escape from ER is prevented by using Brefeldin A, band B increases [a]. [a] Glozman R, Okiyoneda T, Mulvihill CM, Rini JM, Barriere H, Lukacs GL (2009) N-glycans are direct determinants of CFTR folding and stability in secretory and endocytic membrane traffic. J. Cell Biol. 184, 847–862. (TIF) [file pone.0074232.s004.tif]
