# Supplementary material for: Detection of Phospho-Sites Generated by Protein Kinase CK2 in CFTR: Mechanistic Aspects of Thr1471 Phosphorylation
Source: PLoS One. 2013 Sep 18;8(9):e74232. doi: 10.1371/journal.pone.0074232 (PMC3776838; doi:10.1371/journal.pone.0074232)
Supplement: Figure S2 — Annotated fragmentation spectra of the phosphopeptides identified in this study. (PDF) [file pone.0074232.s002.pdf]

MS/MS spectrum of monophosphorylated peptide EGSSDVLVIK phosphorylation site: S1183

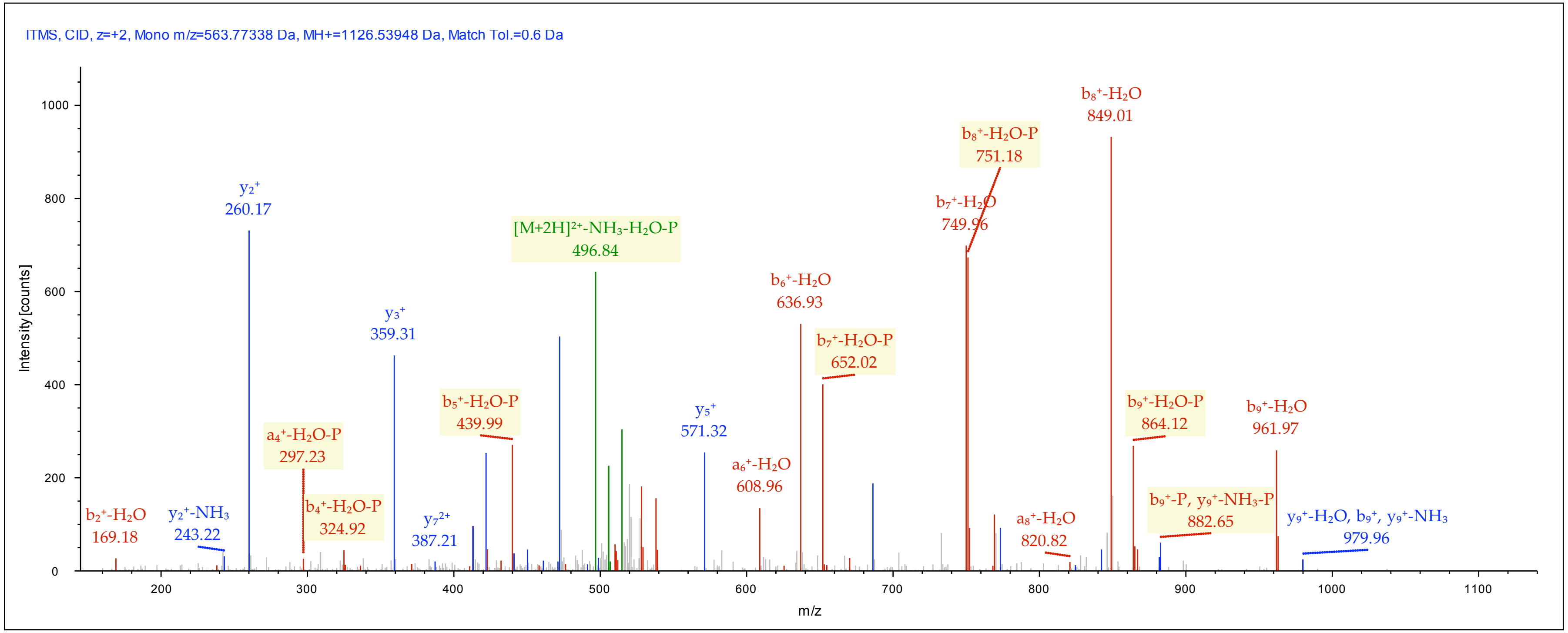

Sequence: EGSSDVLVIK, S3-Phospho (79.96633 Da)
Charge: +2, Monoisotopic m/z: 563.77338 Da (+0.14 mmu/+0.24 ppm), MH+: 1126.53948 Da, RT: 23.56 min,
Identified with: Mascot (v1.27); IonScore:19, Exp Value:1.8E+000, Ions matched by search engine: 5/72
Fragment match tolerance used for search: 0.6 Da
Fragments used for search: a; a-H_2O; b; b-H_2O; y; y-H_2O; y-NH_3

Protein references (1):
- Cystic fibrosis transmembrane conductance regulator OS=Mus musculus GN=Cftr PE=1 SV=2 - [CFTR_MOUSE]

MS/MS spectrum of monophosphorylated peptide EGSSDVLVIKNEHVK phosphorylation sites: S1183 and/or S1184

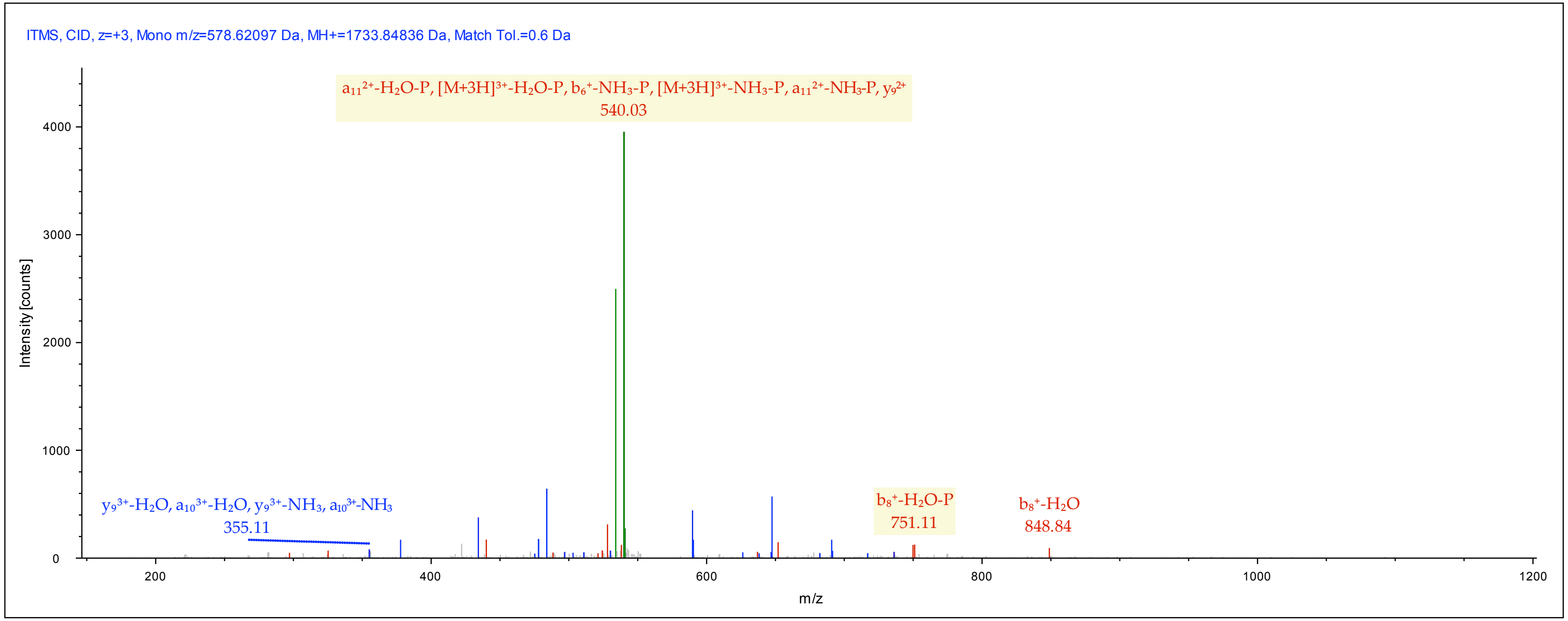

Sequence: EGSSDVLVIKNEHVK, S3- or S4-Phospho (79.96633 Da)
Charge: +3, Monoisotopic m/z: 578.62097 Da (+0.44 mmu/+0.77 ppm), MH+: 1733.84836 Da, RT: 21.32 min,
Identified with: Mascot (v1.27); IonScore:36, Exp Value:4.7E-002, Ions matched by search engine: 8/132
Fragment match tolerance used for search: 0.6 Da
Fragments used for search: a; a-H_2O; a-NH_3; b; b-H_2O; b-NH_3; y; y-H_2O; y-NH_3

Protein references (1):
- Cystic fibrosis transmembrane conductance regulator OS=Mus musculus GN=Cftr PE=1 SV=2 - [CFTR_MOUSE]

MS/MS spectrum of monophosphorylated peptide HHLELSDIYQAPSADSADHLSEK phosphorylation sites: S45 and/or S50

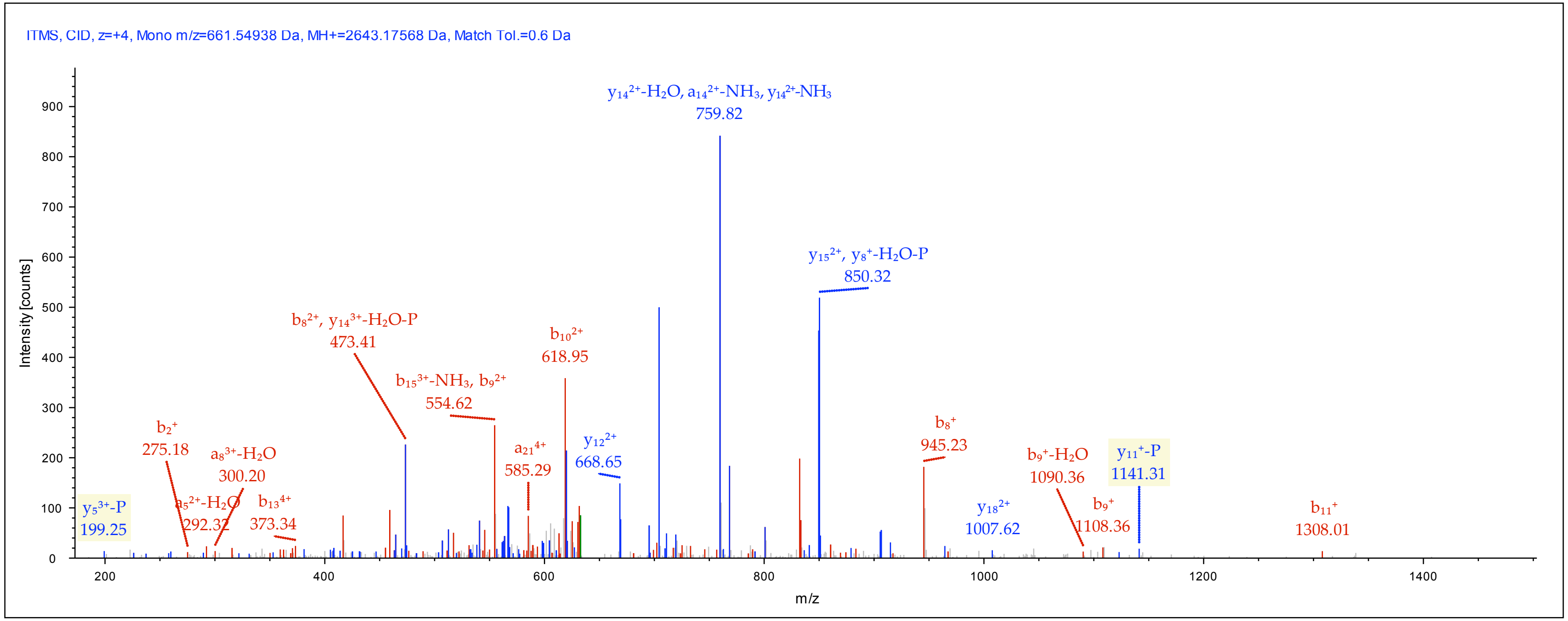

Sequence: HHLELSDIYQAPSADSADHLSEK, S16- or S21-Phospho (79.96633 Da)

Charge: +4, Monoisotopic m/z: 661.54938 Da (+0.83 mmu/+1.26 ppm), MH+: 2643.17568 Da, RT: 23.29 min,

Identified with: SEQUEST (v1.20); XCorr:2.62, Probability:0.00, Ions matched by search engine: 36/195

Fragment match tolerance used for search: 0.6 Da

Fragments used for search: b; b-H_2O; b-NH_3; y; y-H_2O; y-NH_3

Protein references (1):

- Cystic fibrosis transmembrane conductance regulator OS=Mus musculus GN=Cftr PE=1 SV=2 - [CFTR_MOUSE]

MS/MS spectrum of monophosphorylated peptide KISIVQK phosphorylation site: S710

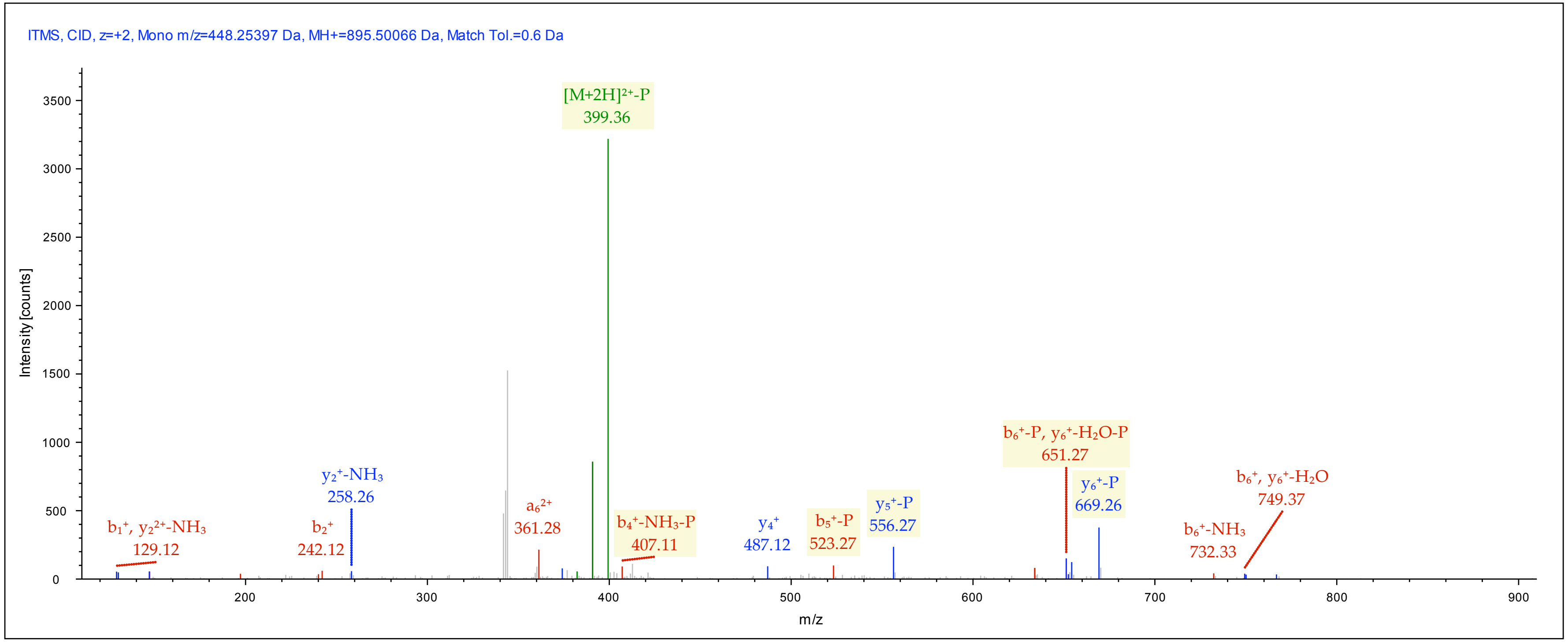

Sequence: KISIVQK, S3-Phospho (79.96633 Da)
Charge: +2, Monoisotopic m/z: 448.25397 Da (-0.31 mmu/-0.7 ppm), MH+: 895.50066 Da, RT: 19.47 min,
Identified with: SEQUEST (v1.20); XCorr:2.69, Probability:0.00, Ions matched by search engine: 16/17
Fragment match tolerance used for search: 0.6 Da
Fragments used for search: b; b-H_2O; b-NH_3; y; y-H_2O; y-NH_3

Protein references (1):
- Cystic fibrosis transmembrane conductance regulator OS=Mus musculus GN=Cftr PE=1 SV=2 - [CFTR_MOUSE]

MS/MS spectrum of monophosphorylated peptide KISLVPQISLNEVDVYSR phosphorylation site: S790

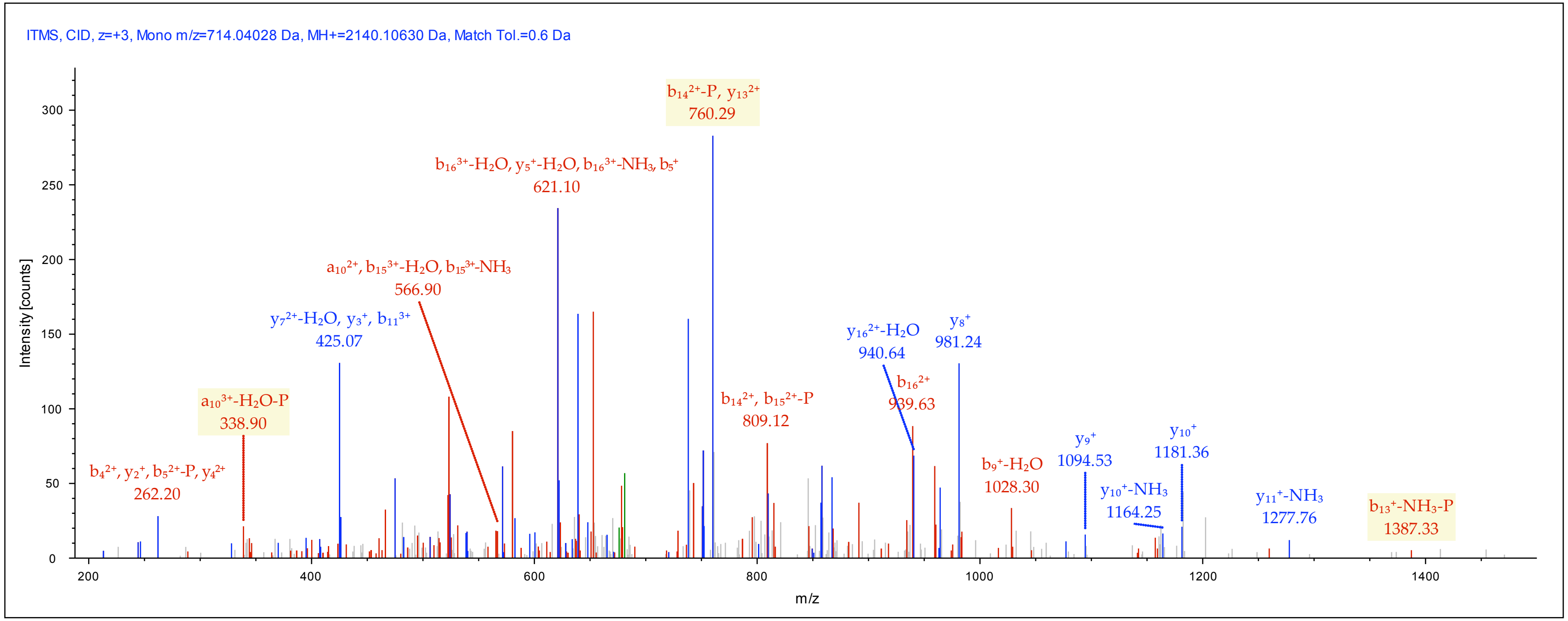

Sequence: KISLVPQISLNEVDVYSR, S3-Phospho (79.96633 Da)
Charge: +3, Monoisotopic m/z: 714.04028 Da (+0.42 mmu/+0.59 ppm), MH+: 2140.10630 Da, RT: 27.27 min,
Identified with: SEQUEST (v1.20); XCorr:4.22, Probability:0.00, Ions matched by search engine: 34/100
Fragment match tolerance used for search: 0.6 Da
Fragments used for search: b; b-H_2O; b-NH_3; y; y-H_2O; y-NH_3

Protein references (1):
- Cystic fibrosis transmembrane conductance regulator OS=Mus musculus GN=Cftr PE=1 SV=2 - [CFTR_MOUSE]

MS/MS spectrum of monophosphorylated peptide KNSILNSFSSVR phosphorylation site: S698

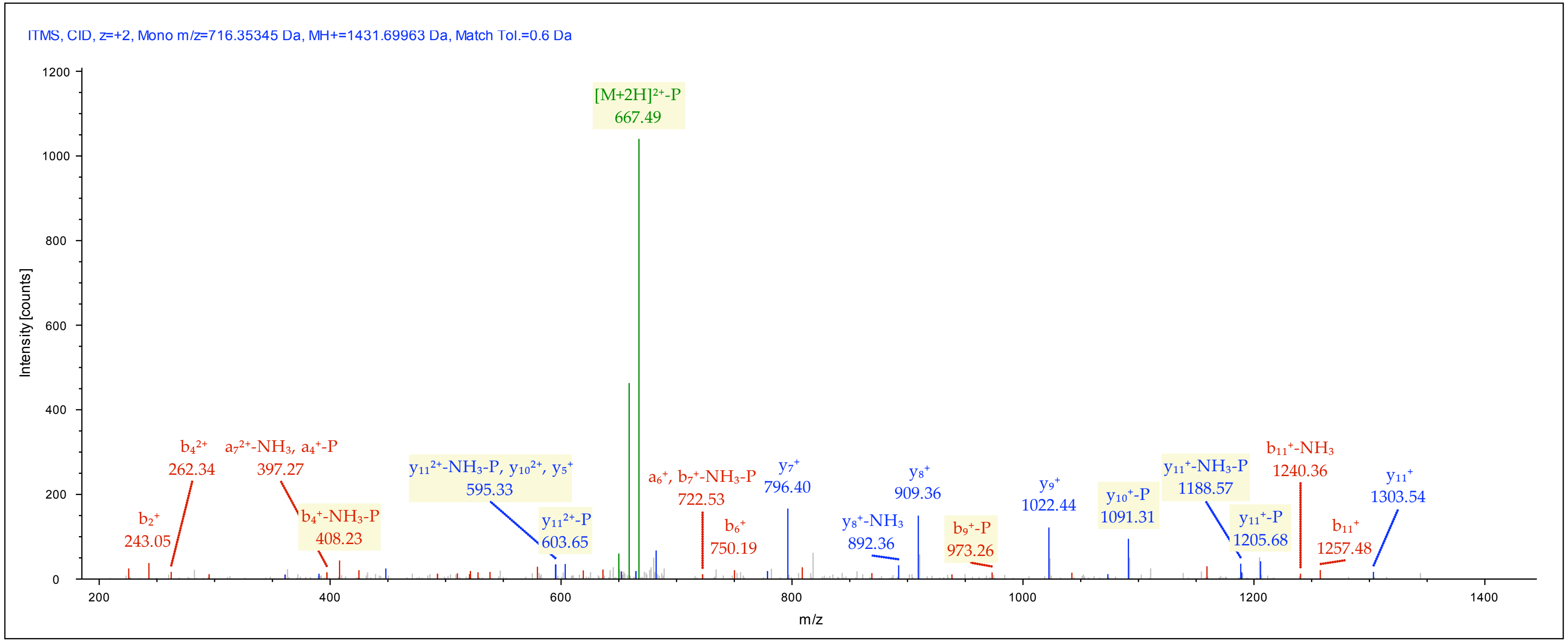

Sequence: KNSILNSFSSVR, S3-Phospho (79.96633 Da)

Charge: +2, Monoisotopic m/z: 716.35345 Da (+0.21 mmu/+0.29 ppm), MH+: 1431.69963 Da, RT: 23.97 min,

Identified with: Mascot (v1.27); IonScore:49, Exp Value:2.5E-003, Ions matched by search engine: 8/132

Fragment match tolerance used for search: 0.6 Da

Fragments used for search: a; a-H_2O; a-NH_3; b; b-H_2O; b-NH_3; y; y-H_2O; y-NH_3

Protein references (1):

- Cystic fibrosis transmembrane conductance regulator OS=Mus musculus GN=Cftr PE=1 SV=2 - [CFTR_MOUSE]

MS/MS spectrum of monophosphorylated peptide RKNSILNSFSSVR phosphorylation site: S698

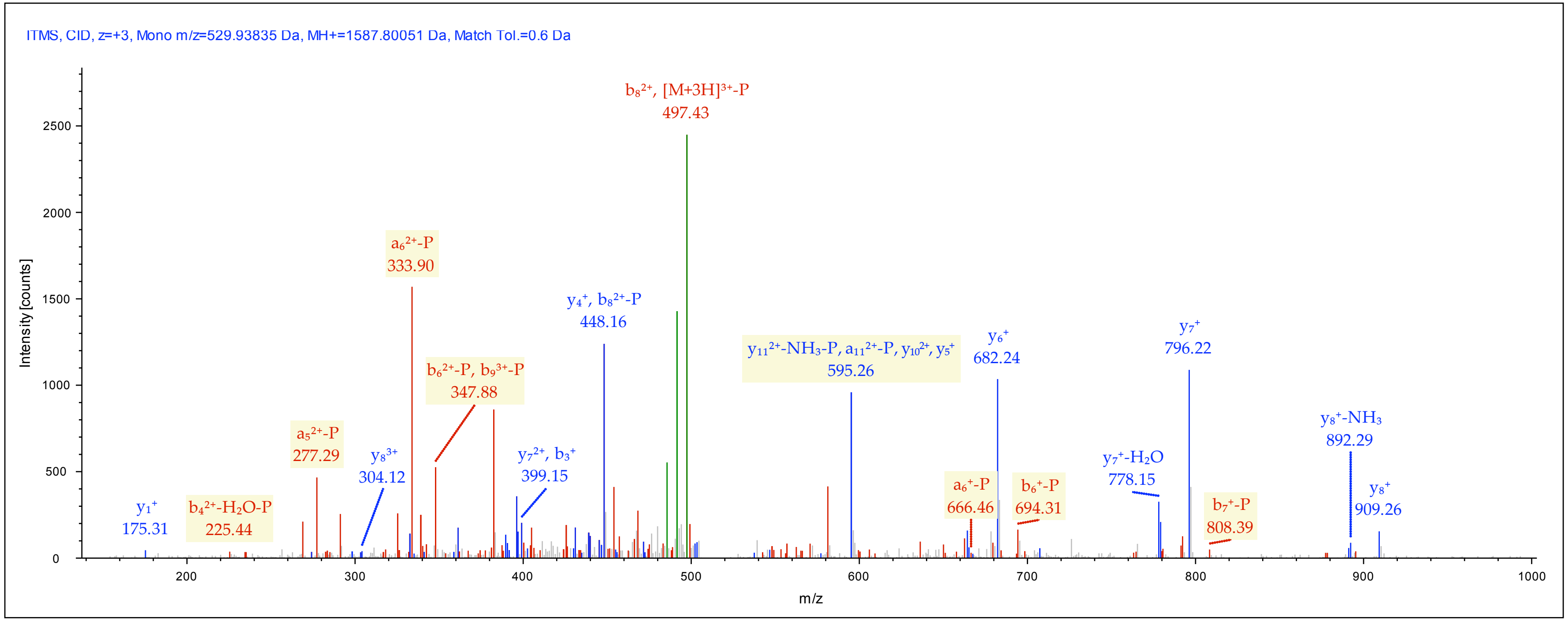

Sequence: RKNSILNSFSSVR, S4-Phospho (79.96633 Da)
Charge: +3, Monoisotopic m/z: 529.93835 Da (+0.06 mmu/+0.11 ppm), MH+: 1587.80051 Da, RT: 22.57 min,
Identified with: SEQUEST (v1.20); XCorr:3.06, Probability:0.00, Ions matched by search engine: 30/70
Fragment match tolerance used for search: 0.6 Da
Fragments used for search: b; b-H_2O; b-NH_3; y; y-H_2O; y-NH_3

Protein references (1):
- Cystic fibrosis transmembrane conductance regulator OS=Mus musculus GN=Cftr PE=1 SV=2 - [CFTR_MOUSE]

MS/MS spectrum of monophosphorylated peptide LSLVPDSEQGEAALPR phosphorylation site: S737

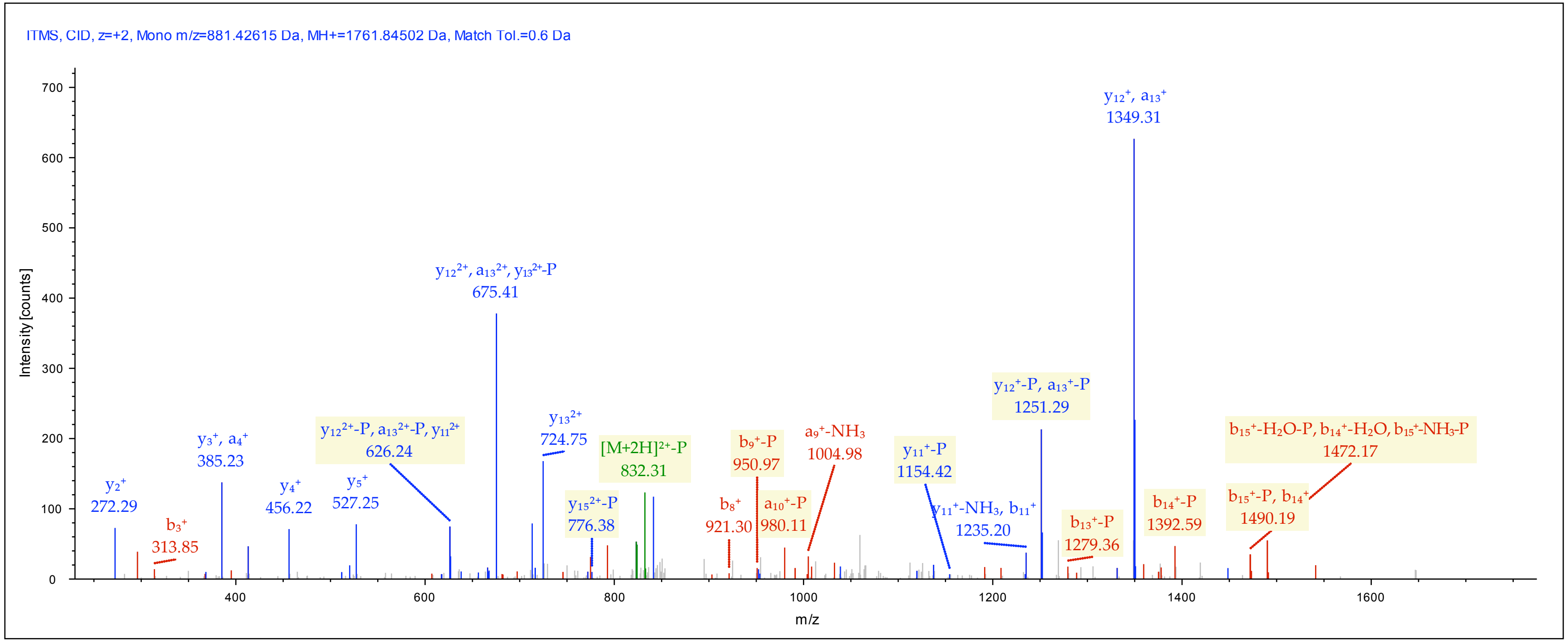

Sequence: LSLVPDSEQGEAALPR, S7-Phospho (79.96633 Da)
Charge: +2, Monoisotopic m/z: 881.42615 Da (+1.53 mmu/+1.73 ppm), MH+: 1761.84502 Da, RT: 25.14 min,
Identified with: SEQUEST (v1.20); XCorr:2.81, Probability:0.00, Ions matched by search engine: 28/44
Fragment match tolerance used for search: 0.6 Da
Fragments used for search: b; b-H_2O; b-NH_3; y; y-H_2O; y-NH_3

Protein references (1):
- Cystic fibrosis transmembrane conductance regulator OS=Mus musculus GN=Cftr PE=1 SV=2 - [CFTR_MOUSE]

MS/MS spectrum of monophosphorylated peptide RLSLVPDSEQGEAALPR phosphorylation site: S732

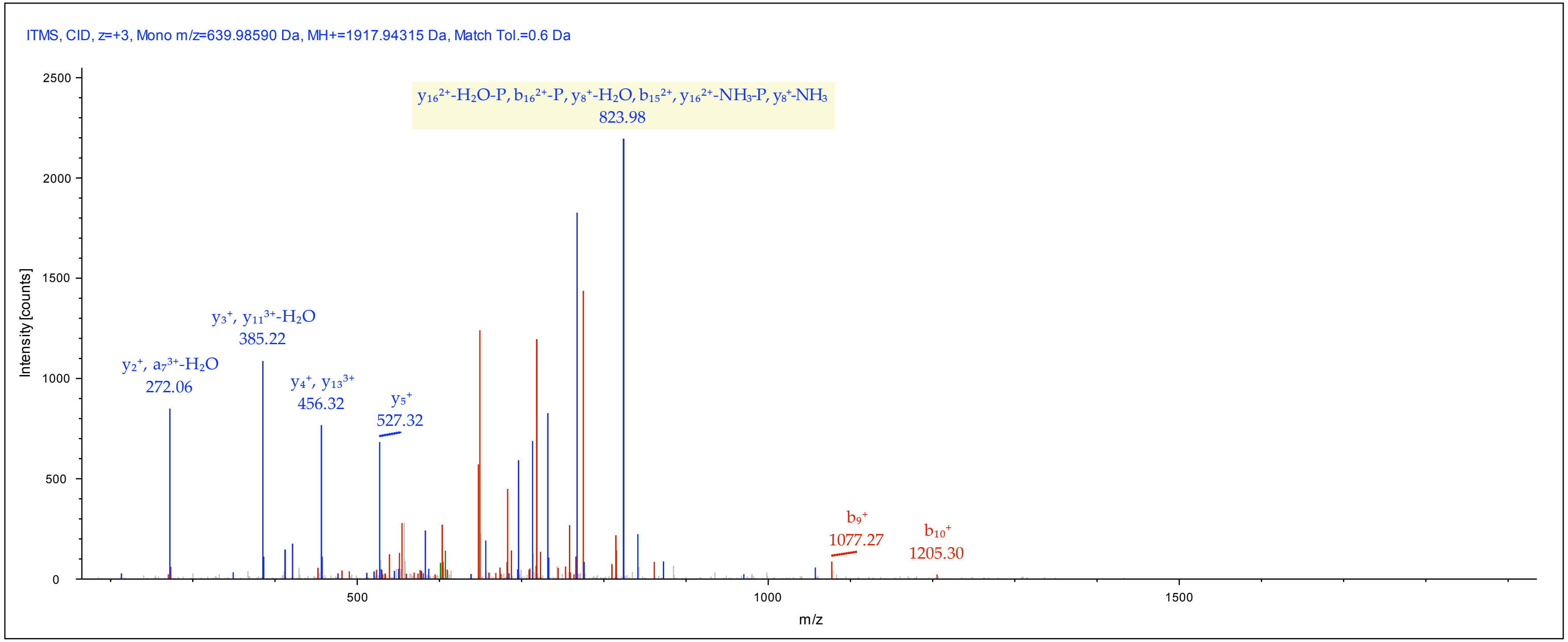

Sequence: RLSLVPDSEQGEAALPR, S3-Phospho (79.96633 Da)
Charge: +3, Monoisotopic m/z: 639.98590 Da (+0.02 mmu/+0.03 ppm), MH+: 1917.94315 Da, RT: 24.06 min,
Identified with: Mascot (v1.27); IonScore:40, Exp Value:2.0E-002, Ions matched by search engine: 9/192
Fragment match tolerance used for search: 0.6 Da
Fragments used for search: a; a-H_2O; a-NH_3; b; b-H_2O; b-NH_3; y; y-H_2O; y-NH_3

Protein references (1):
- Cystic fibrosis transmembrane conductance regulator OS=Mus musculus GN=Cftr PE=1 SV=2 - [CFTR_MOUSE]

MS/MS spectrum of monophosphorylated peptide RLSQDSTLNITEEINEEDLK phosphorylation site: S808

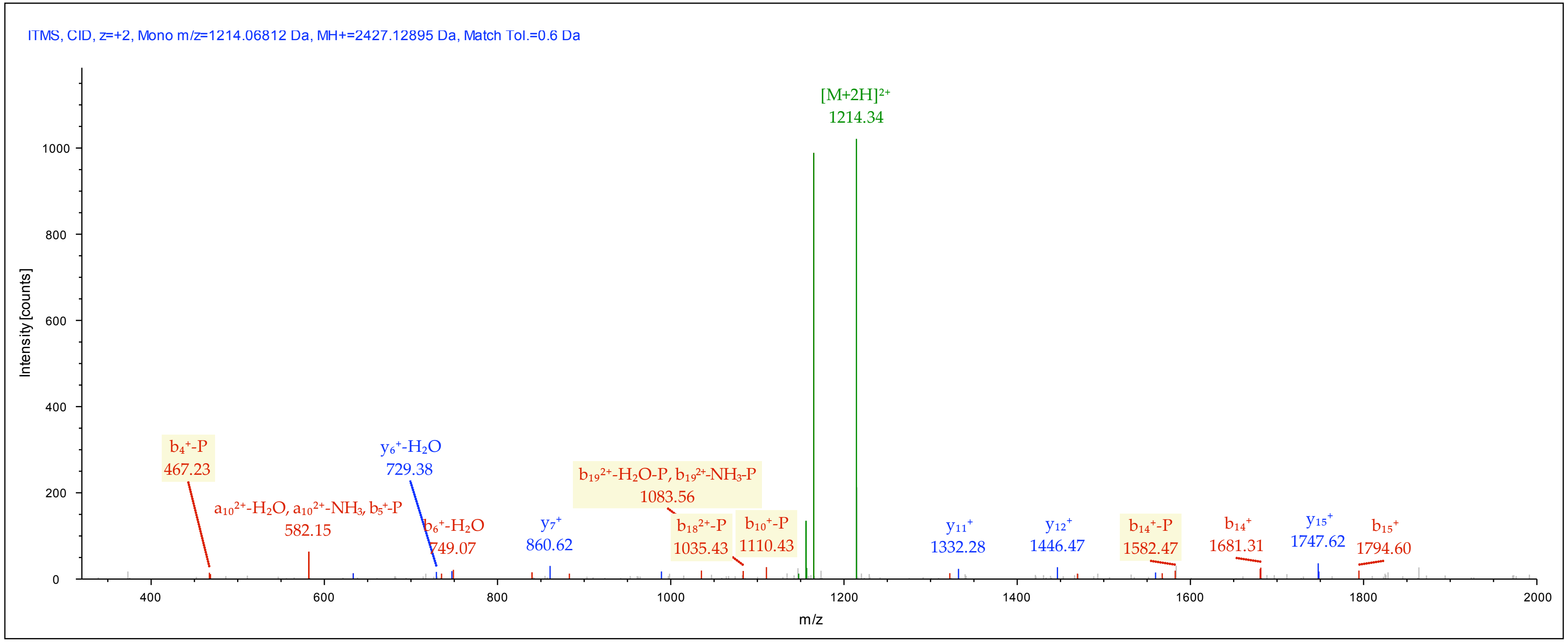

Sequence: RLSQDSTLNITEEINEEDLK, S3-Phospho (79.96633 Da)
Charge: +2, Monoisotopic m/z: 1214.06812 Da (+0.09 mmu/+0.07 ppm), MH+: 2427.12895 Da, RT: 25.42 min,
Identified with: Mascot (v1.27); IonScore:49, Exp Value:3.4E-003, Ions matched by search engine: 17/228
Fragment match tolerance used for search: 0.6 Da
Fragments used for search: a; a-H_2O; a-NH_3; b; b-H_2O; b-NH_3; y; y-H_2O; y-NH_3

Protein references (2):
- Cystic fibrosis transmembrane conductance regulator OS=Mus musculus GN=Cftr PE=1 SV=2 - [CFTR_MOUSE]

MS/MS spectrum of monophosphorylated peptide RLSQDSTLNITEEINEEDLK phosphorylation site: S808

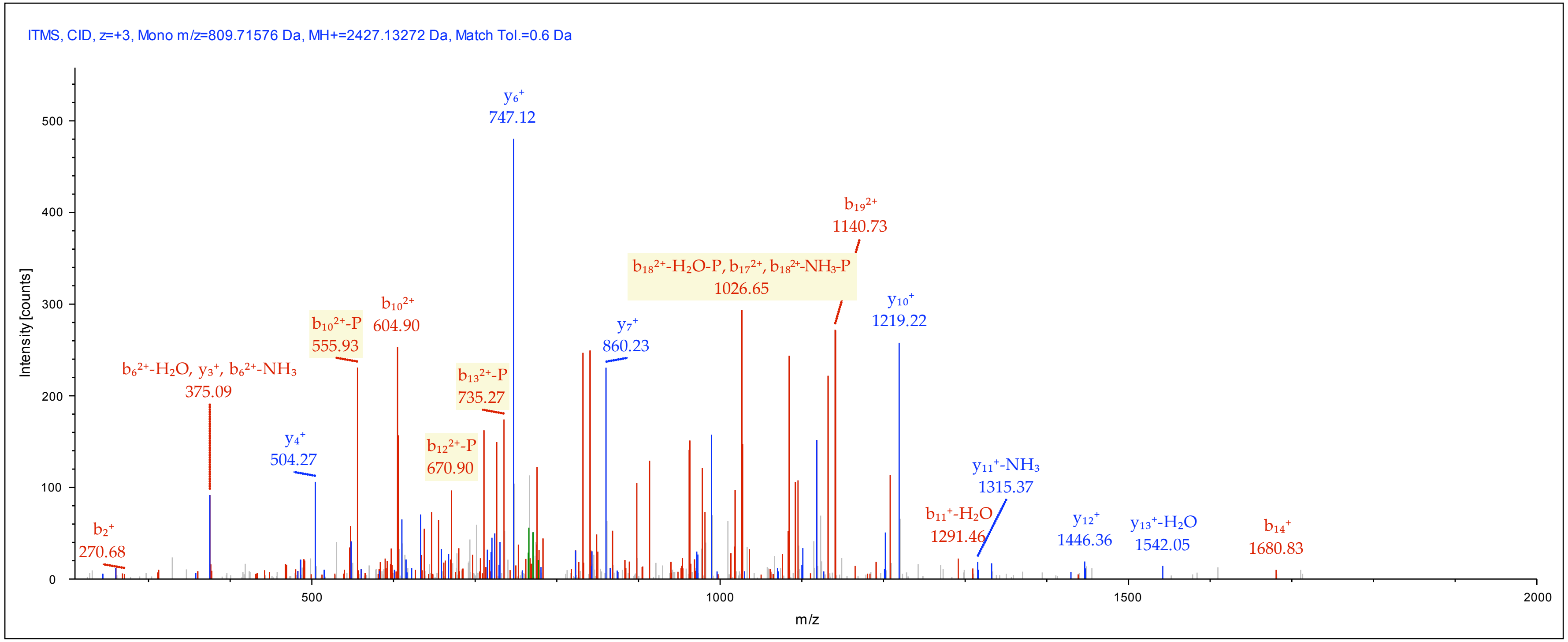

Sequence: RLSQDSTLNITEEINEEDLK, S3-Phospho (79.96633 Da)
Charge: +3, Monoisotopic m/z: 809.71576 Da (+1.32 mmu/+1.63 ppm), MH+: 2427.13272 Da, RT: 25.41 min,
Identified with: Mascot (v1.27); IonScore:50, Exp Value:2.8E-003, Ions matched by search engine: 11/228
Fragment match tolerance used for search: 0.6 Da
Fragments used for search: a; a-H_2O; a-NH_3; b; b-H_2O; b-NH_3; y; y-H_2O; y-NH_3

Protein references (2):
- Cystic fibrosis transmembrane conductance regulator OS=Mus musculus GN=Cftr PE=1 SV=2 - [CFTR_MOUSE]

MS/MS spectrum of bis-phosphorylated peptide RLSQDSTLNITEEINEEDLK phosphorylation sites: S808 and S811

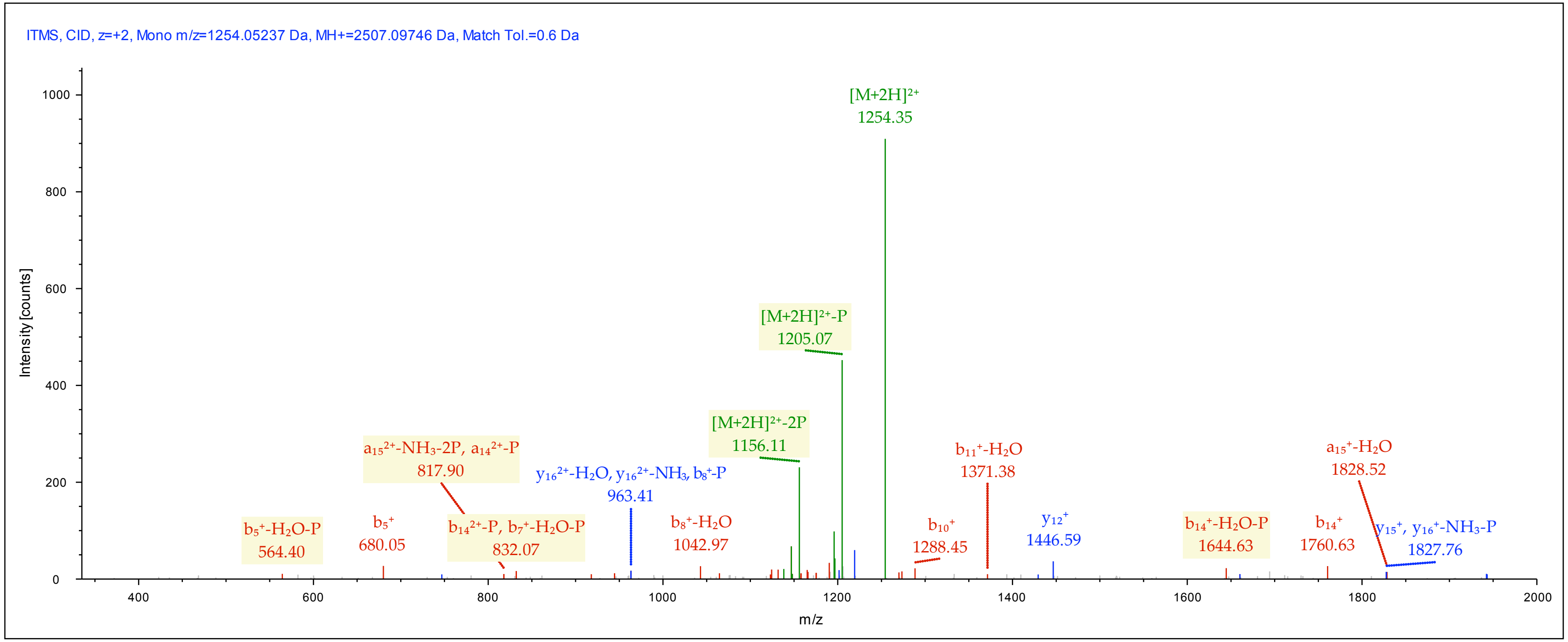

Sequence: RLSQDSTLNITEEINEEDLK, S3-Phospho (79.96633 Da) and S6-Phospho (79.96633 Da)
Charge: +2, Monoisotopic m/z: 1254.05237 Da (+1.18 mmu/+0.94 ppm), MH+: 2507.09746 Da, RT: 25.89 min,
Identified with: Mascot (v1.27); IonScore:23, Exp Value:1.4E+000, Ions matched by search engine: 15/228
Fragment match tolerance used for search: 0.6 Da
Fragments used for search: a; a-H_2O; a-NH_3; b; b-H_2O; b-NH_3; y; y-H_2O; y-NH_3

Protein references (2):
- Cystic fibrosis transmembrane conductance regulator OS=Mus musculus GN=Cftr PE=1 SV=2 - [CFTR_MOUSE]

MS/MS spectrum of bis-phosphorylated peptide RLSQDSTLNITEEINEEDLK phosphorylation sites: S808 and S811

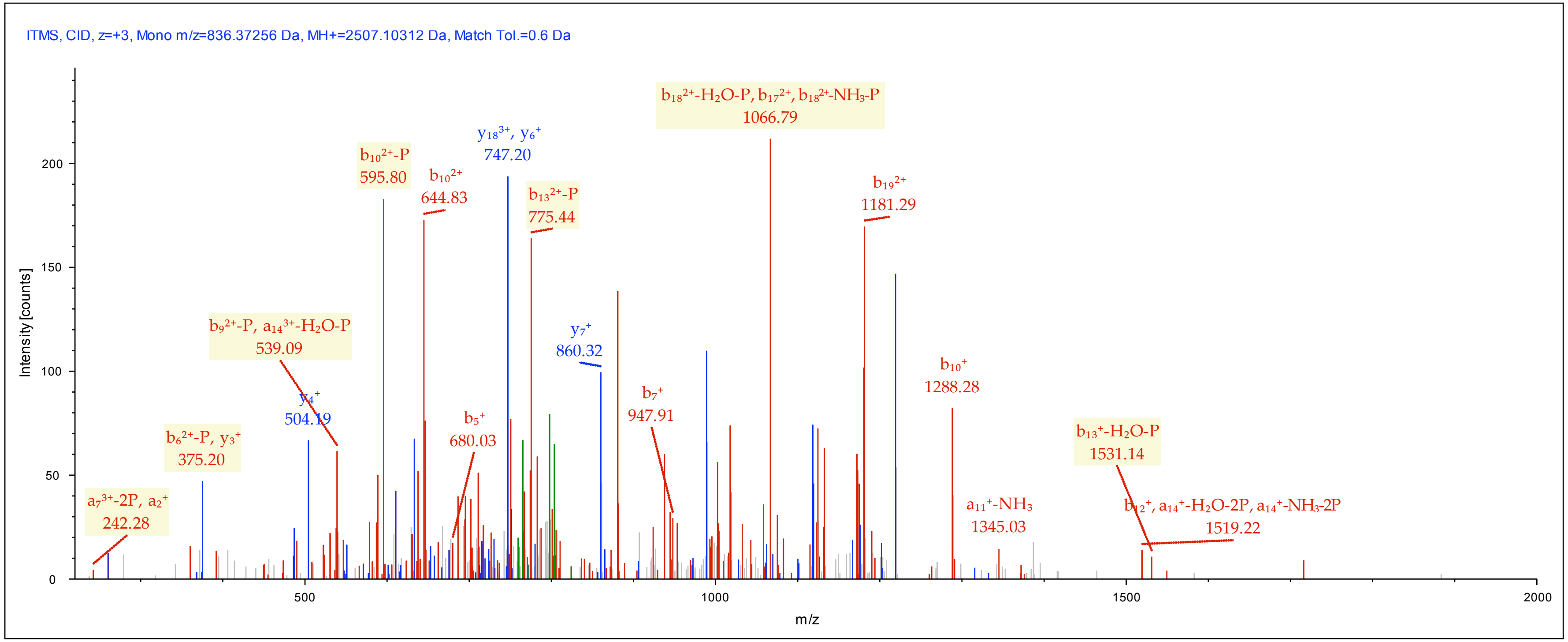

Sequence: RLSQDSTLNITEEINEEDLK, S3-Phospho (79.96633 Da) and S6-Phospho (79.96633 Da)
Charge: +3, Monoisotopic m/z: 836.37256 Da (+2.68 mmu/+3.2 ppm), MH+: 2507.10312 Da, RT: 25.89 min,
Identified with: Mascot (v1.27); IonScore:45, Exp Value:9.6E-003, Ions matched by search engine: 9/228
Fragment match tolerance used for search: 0.6 Da
Fragments used for search: a; a-H_2O; a-NH_3; b; b-H_2O; b-NH_3; y; y-H_2O; y-NH_3

Protein references (2):
- Cystic fibrosis transmembrane conductance regulator OS=Mus musculus GN=Cftr PE=1 SV=2 - [CFTR_MOUSE]

MS/MS spectrum of bis-phosphorylated peptide RFSVDDSSAPWSKPK phosphorylation site: S670

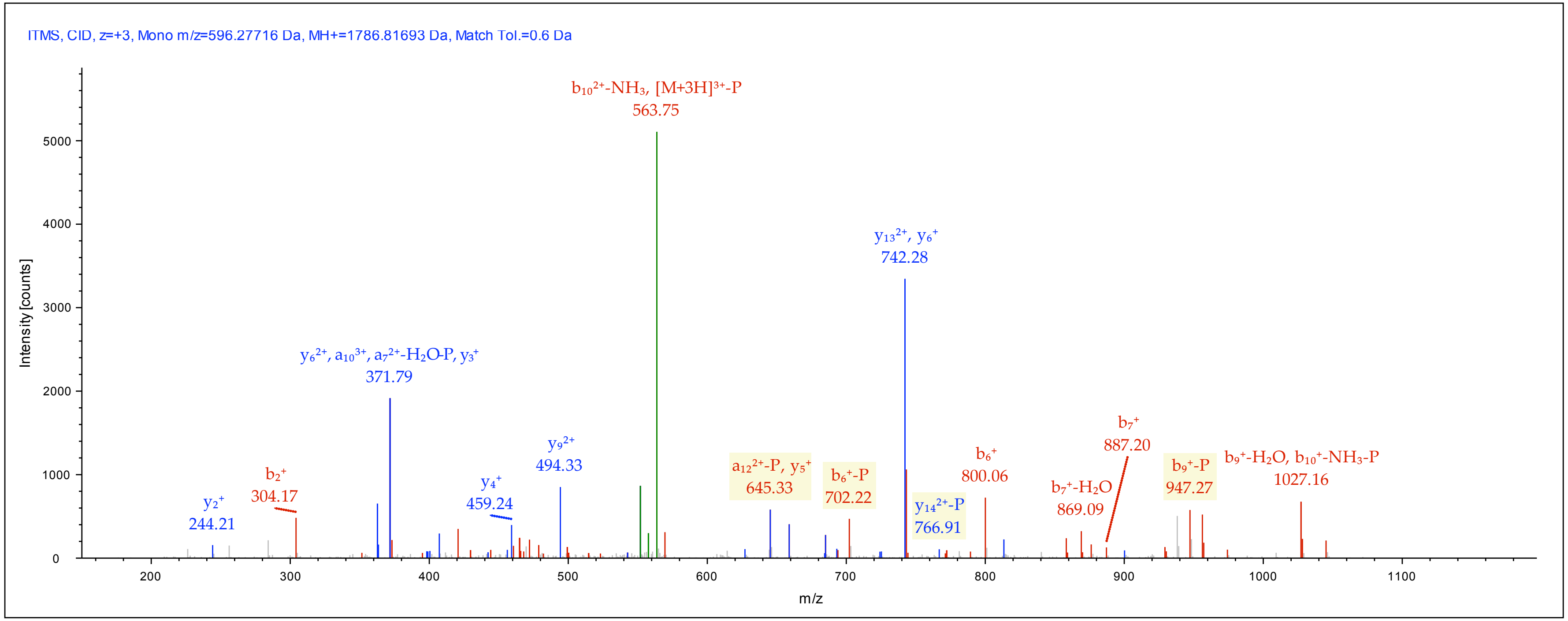

Sequence: RFSVDDSSAPWSKPK, S3-Phospho (79.96633 Da)

Charge: +3, Monoisotopic m/z: 596.27716 Da (+0.29 mmu/+0.48 ppm), MH+: 1786.81693 Da, RT: 22.52 min,

Identified with: Mascot (v1.27); IonScore:32, Exp Value:1.3E-001, Ions matched by search engine: 17/168

Fragment match tolerance used for search: 0.6 Da

Fragments used for search: a; a-H_2O; a-NH_3; b; b-H_2O; b-NH_3; y; y-H_2O; y-NH_3

Protein references (1):

- Cystic fibrosis transmembrane conductance regulator OS=Mus musculus GN=Cftr PE=1 SV=2 - [CFTR_MOUSE]

MS/MS spectrum of monophosphorylated peptide RQSVLDLMTFTPNSGSSNLQR phosphorylation site: S763

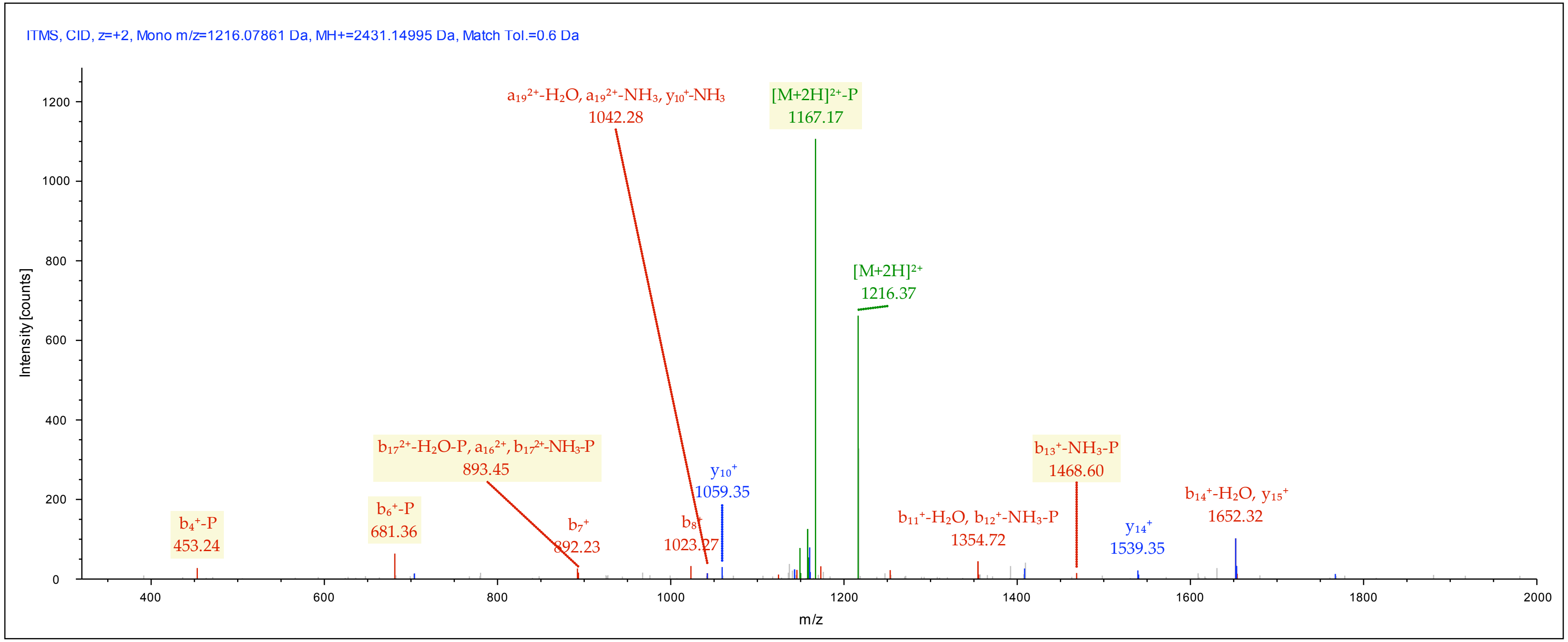

Sequence: RQSVLDLMTFTPNSGSSNLQR, S3-Phospho (79.96633 Da)
Charge: +2, Monoisotopic m/z: 1216.07861 Da (+3.15 mmu/+2.59 ppm), MH+: 2431.14995 Da, RT: 26.32 min,
Identified with: Mascot (v1.27); IonScore:54, Exp Value:1.1E-003, Ions matched by search engine: 10/240
Fragment match tolerance used for search: 0.6 Da
Fragments used for search: a; a-H_2O; a-NH_3; b; b-H_2O; b-NH_3; y; y-H_2O; y-NH_3

Protein references (1):
- Cystic fibrosis transmembrane conductance regulator OS=Mus musculus GN=Cftr PE=1 SV=2 - [CFTR_MOUSE]

MS/MS spectrum of monophosphorylated peptide RQSVLDLMTFTPNSGSSNLQR phosphorylation site: S763

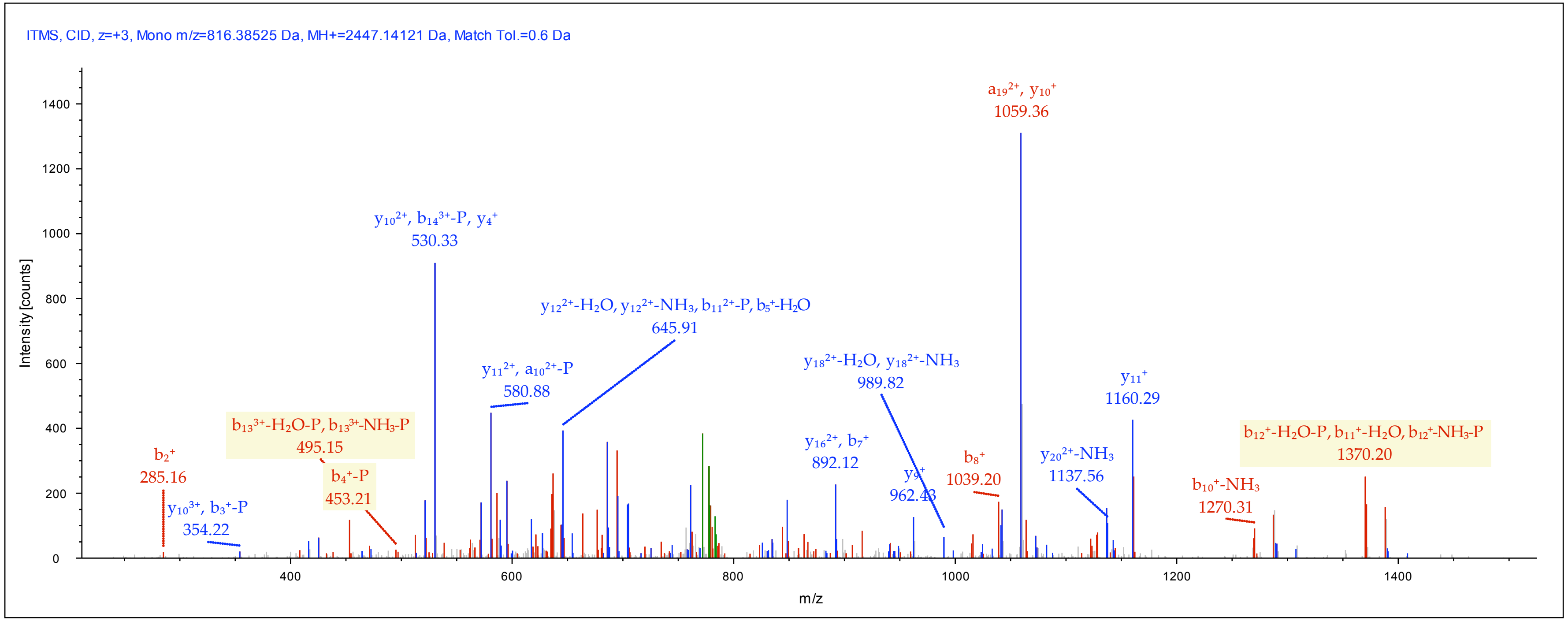

Sequence: RQSVLDLMTFTPNSGSSNLQR, S3-Phospho (79.96633 Da), M8-Oxidation (15.99492 Da)
Charge: +3, Monoisotopic m/z: 816.38525 Da (+0.88 mmu/+1.08 ppm), MH+: 2447.14121 Da, RT: 24.66 min,
Identified with: Mascot (v1.27); IonScore:42, Exp Value:1.9E-002, Ions matched by search engine: 11/240
Fragment match tolerance used for search: 0.6 Da
Fragments used for search: a; a-H_2O; a-NH_3; b; b-H_2O; b-NH_3; y; y-H_2O; y-NH_3

Protein references (1):
- Cystic fibrosis transmembrane conductance regulator OS=Mus musculus GN=Cftr PE=1 SV=2 - [CFTR_MOUSE]

MS/MS spectrum of monophosphorylated peptide RRQSVLDLMTFTPNSGSSNLQR phosphorylation site: S763

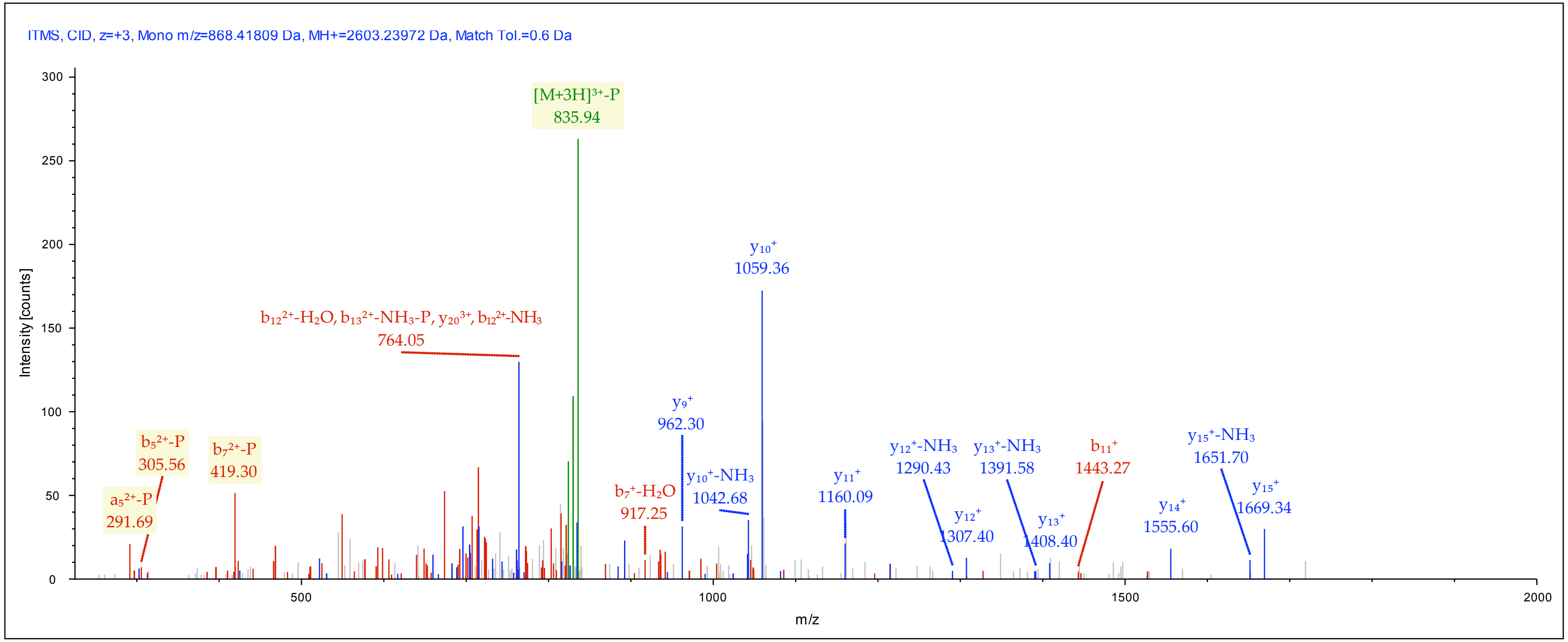

Sequence: RRQSVLDLMTFTPNSGSSNLQR, S4-Phospho (79.96633 Da), M9-Oxidation (15.99492 Da)
Charge: +3, Monoisotopic m/z: 868.41809 Da (+0.01 mmu/+0.01 ppm), MH+: 2603.23972 Da, RT: 23.76 min,
Identified with: SEQUEST (v1.20); XCorr:3.25, Probability:0.00, Ions matched by search engine: 34/124
Fragment match tolerance used for search: 0.6 Da
Fragments used for search: b; b-H_2O; b-NH_3; y; y-H_2O; y-NH_3

Protein references (1):
- Cystic fibrosis transmembrane conductance regulator OS=Mus musculus GN=Cftr PE=1 SV=2 - [CFTR_MOUSE]

MS/MS spectrum of monophosphorylated peptide SSILTETLR phosphorylation sites: S659 and/or S660

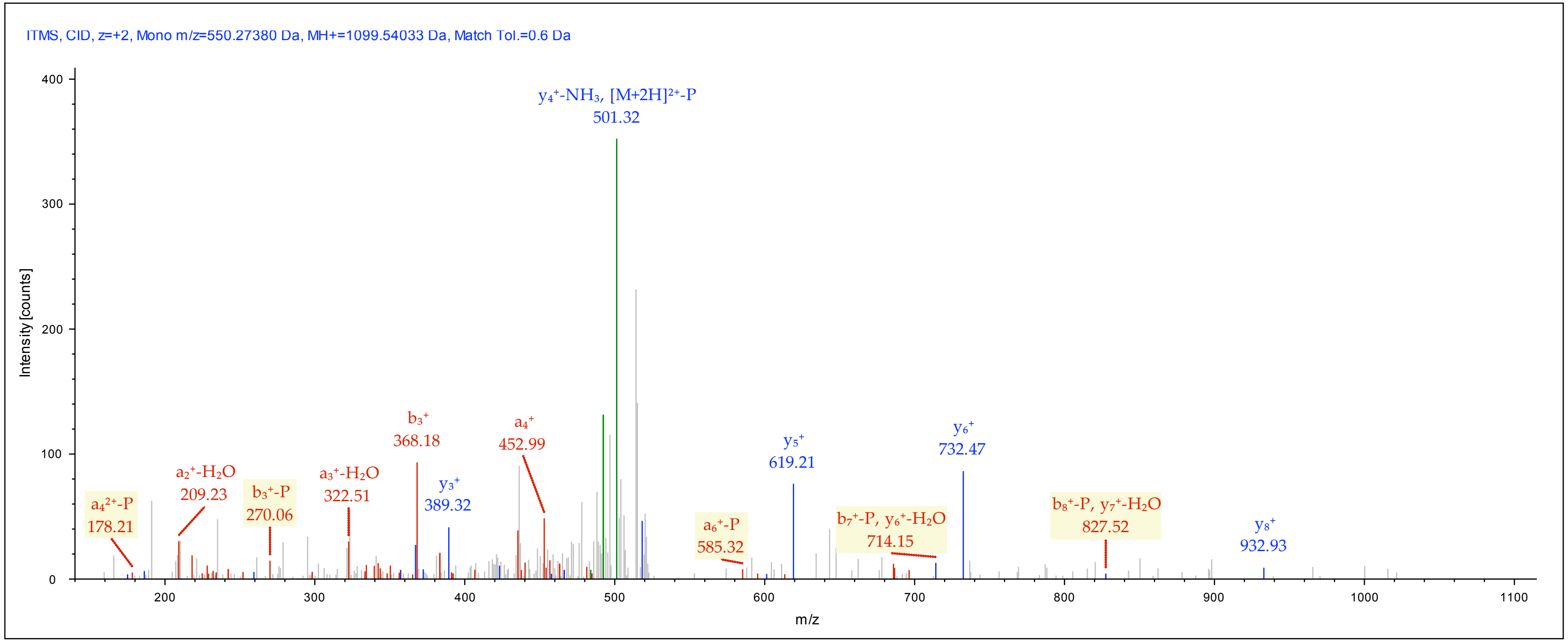

Sequence: SSILTETLR, S1- or S2-Phospho (79.96633 Da)
Charge: +2, Monoisotopic m/z: 550.27380 Da (+0.4 mmu/+0.74 ppm), MH+: 1099.54033 Da, RT: 25.19 min,
Identified with: SEQUEST (v1.20); XCorr:0.85, Probability:0.00, Ions matched by search engine: 11/32
Fragment match tolerance used for search: 0.6 Da
Fragments used for search: b; b-H_2O; y; y-H_2O; y-NH_3

Protein references (1):
- Cystic fibrosis transmembrane conductance regulator OS=Mus musculus GN=Cftr PE=1 SV=2 - [CFTR_MOUSE]

MS/MS spectrum of monophosphorylated peptide RSSILTETLR phosphorylation sites: S659 and/or S660

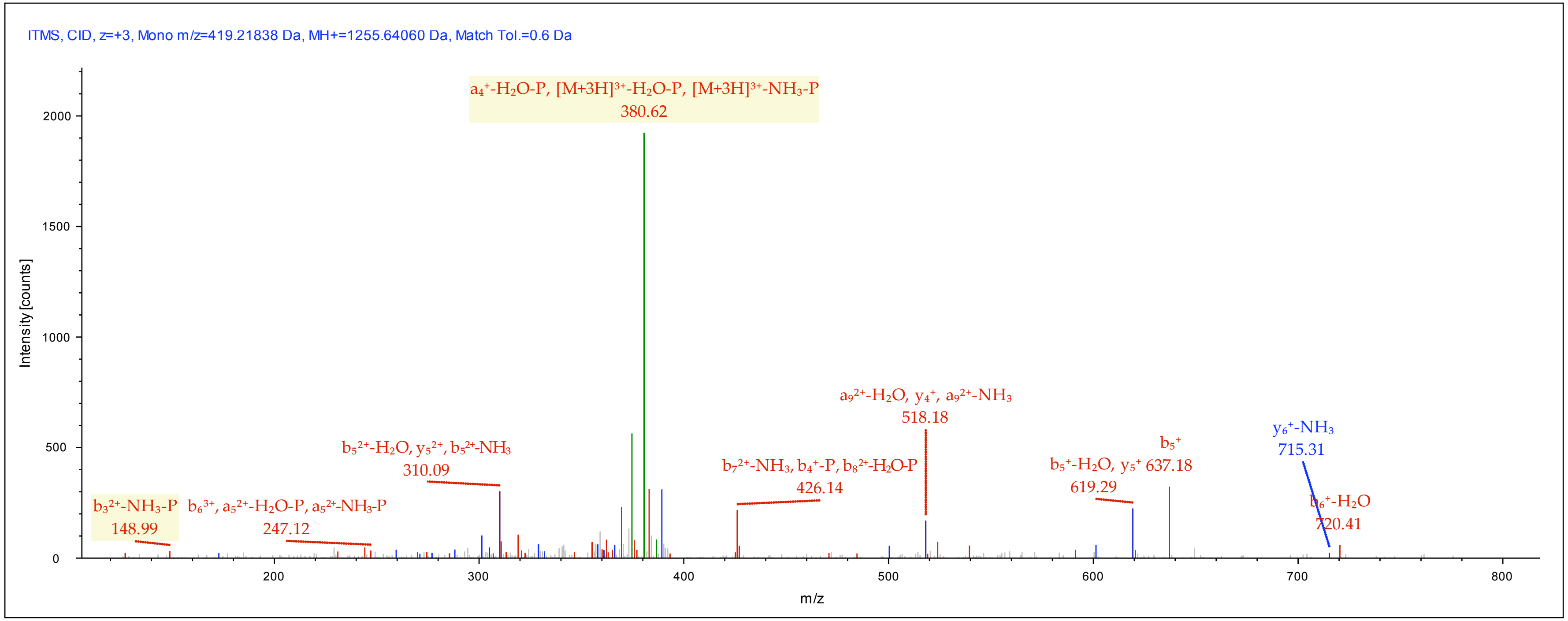

Sequence: RSSILTETLR, S2- or S3-Phospho (79.96633 Da)

Charge: +3, Monoisotopic m/z: 419.21838 Da (-0.01 mmu/-0.04 ppm), MH+: 1255.64060 Da, RT: 22.54 min,

Identified with: SEQUEST (v1.20); XCorr:1.71, Probability:0.00, Ions matched by search engine: 16/52

Fragment match tolerance used for search: 0.6 Da

Fragments used for search: b; b-H_2O; b-NH_3; y; y-H_2O; y-NH_3

Protein references (1):

- Cystic fibrosis transmembrane conductance regulator OS=Mus musculus GN=Cftr PE=1 SV=2 - [CFTR_MOUSE]

MS/MS spectrum of monophosphorylated peptide RSSILTETLR phosphorylation site: T665

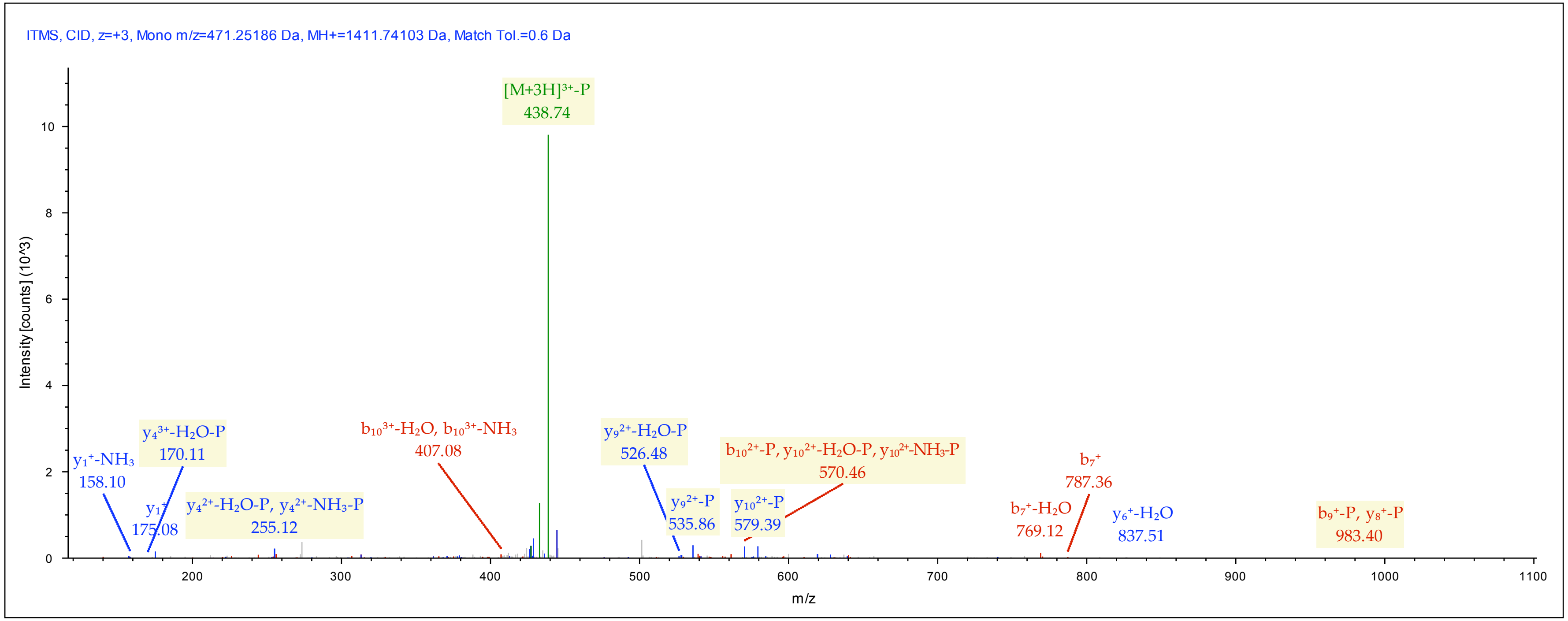

Sequence: RSSILTETLRR, T8-Phospho (79.96633 Da)

Charge: +3, Monoisotopic m/z: 471.25186 Da (-0.24 mmu/-0.52 ppm), MH+: 1411.74103 Da, RT: 21.62 min,

Identified with: SEQUEST (v1.20); XCorr:1.68, Probability:0.00, Ions matched by search engine: 22/58

Fragment match tolerance used for search: 0.6 Da

Fragments used for search: b; b-H_2O; b-NH_3; y; y-H_2O; y-NH_3

Protein references (1):

- Cystic fibrosis transmembrane conductance regulator OS=Mus musculus GN=Cftr PE=1 SV=2 - [CFTR_MOUSE]

MS/MS spectrum of monophosphorylated peptide SIFQQAISSSEK phosphorylation site: S1439

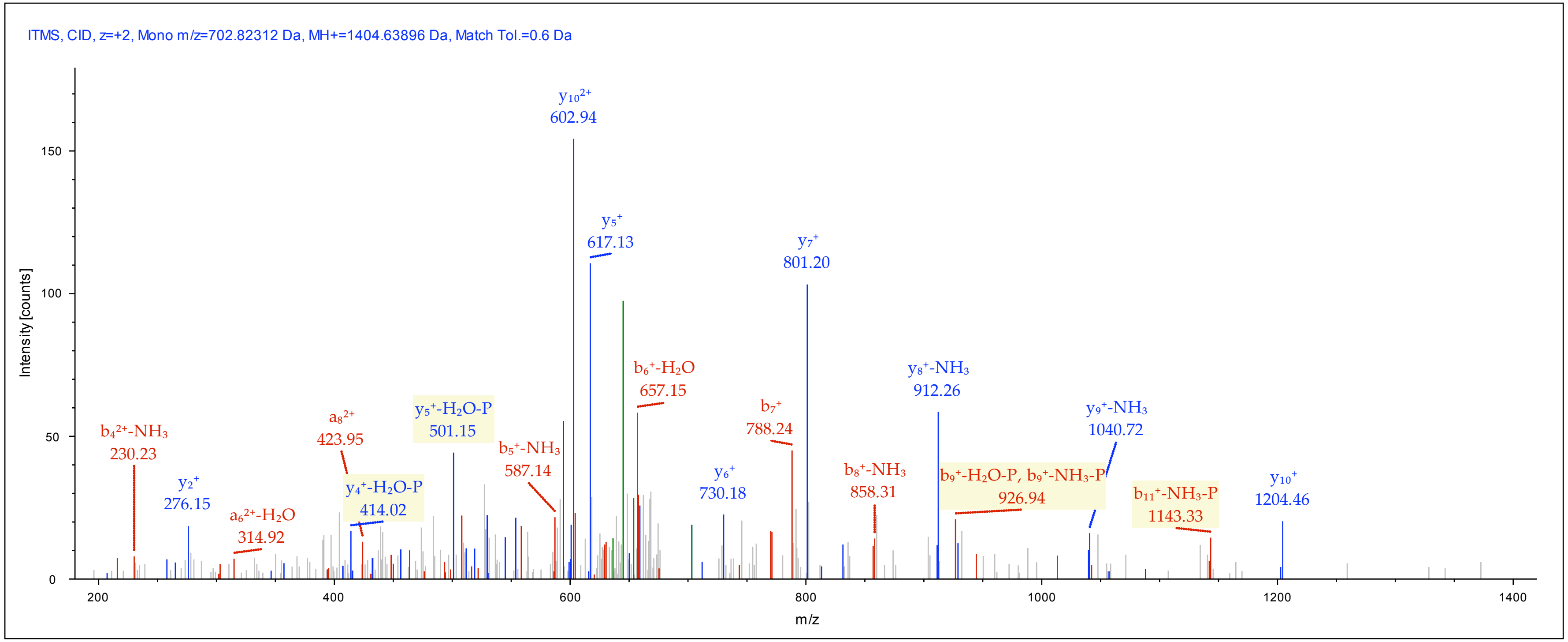

Sequence: SIFQQAISSSEK, S9-Phospho (79.96633 Da)
Charge: +2, Monoisotopic m/z: 702.82312 Da (-0.87 mmu/-1.24 ppm), MH+: 1404.63896 Da, RT: 23.78 min,
Identified with: Mascot (v1.27); IonScore:13, Exp Value:9.0E+000, Ions matched by search engine: 7/120
Fragment match tolerance used for search: 0.6 Da
Fragments used for search: a; a-H_2O; a-NH_3; b; b-H_2O; b-NH_3; y; y-H_2O; y-NH_3

Protein references (1):
- Cystic fibrosis transmembrane conductance regulator OS=Mus musculus GN=Cftr PE=1 SV=2 - [CFTR_MOUSE]

MS/MS spectrum of monophosphorylated peptide SIFQQAISSSEK phosphorylation site: S1440

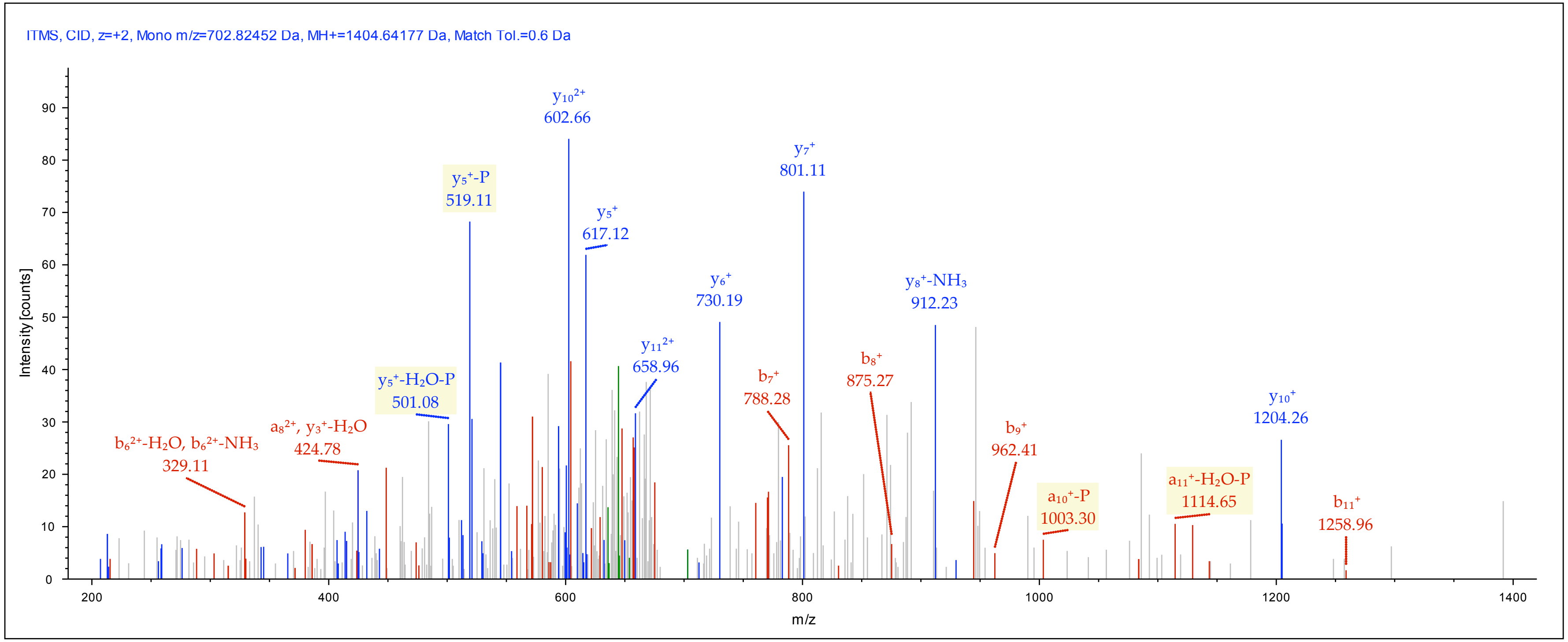

Sequence: SIFQQAISSSEK, S10-Phospho (79.96633 Da)

Charge: +2,   Monoisotopic m/z: 702.82452 Da (+0.53 mmu/+0.76 ppm),   MH+: 1404.64177 Da,   RT: 23.72 min,

Identified with: SEQUEST (v1.20); XCorr:2.00, Probability:0.00, Ions matched by search engine: 11/32

Fragment match tolerance used for search: 0.6 Da

Fragments used for search: b; b-H_2O; b-NH_3; y; y-H_2O; y-NH_3

Protein references (1):

- Cystic fibrosis transmembrane conductance regulator OS=Mus musculus GN=Cftr PE=1 SV=2 - [CFTR_MOUSE]

MS/MS spectrum of monophosphorylated peptide TPLCIDGESDDLQEK phosphorylation site: S723

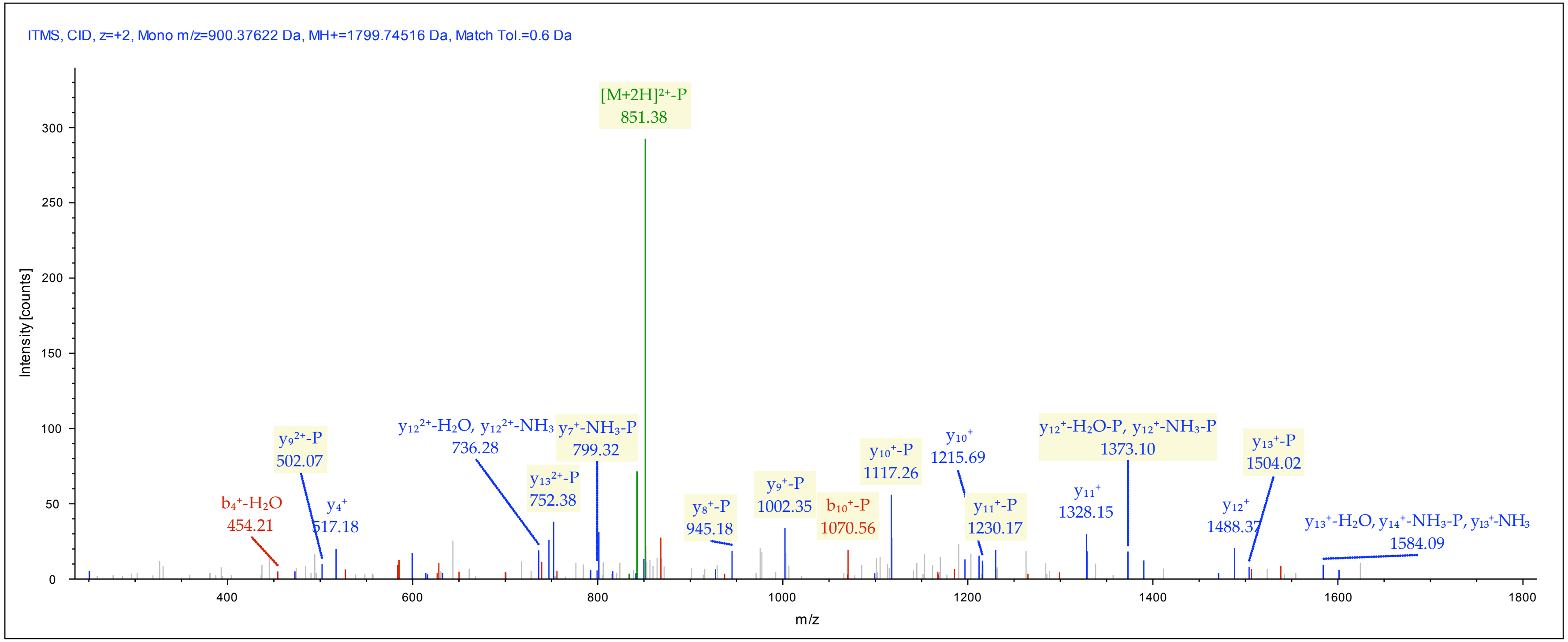

Sequence: TPLCIDGESDDLQEK, C4-Carbamidomethyl (57.02146 Da), S9-Phospho (79.96633 Da)
Charge: +2, Monoisotopic m/z: 900.37622 Da (+2.29 mmu/+2.54 ppm), MH+: 1799.74516 Da, RT: 23.33 min,
Identified with: Mascot (v1.27); IonScore:35, Exp Value:5.2E-002, Ions matched by search engine: 8/120
Fragment match tolerance used for search: 0.6 Da
Fragments used for search: a; a-H_2O; a-NH_3; b; b-H_2O; b-NH_3; y; y-H_2O; y-NH_3

Protein references (1):
- Cystic fibrosis transmembrane conductance regulator OS=Mus musculus GN=Cftr PE=1 SV=2 - [CFTR_MOUSE]

MS/MS spectrum of monophosphorylated peptide TPLCIDGESDDLQEKR phosphorylation site: S723

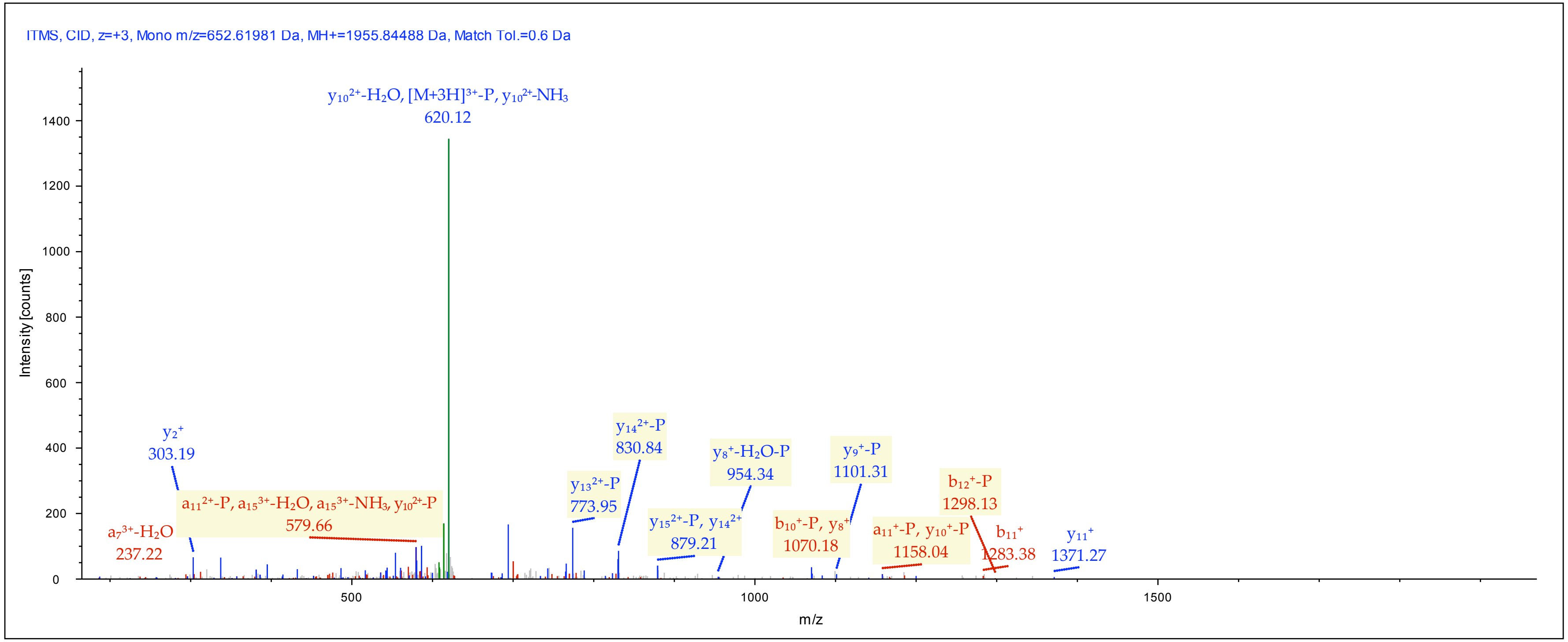

Sequence: TPLCIDGESDDLQEKR, C4-Carbamidomethyl (57.02146 Da), S9-Phospho (79.96633 Da)

Charge: +3, Monoisotopic m/z: 652.61981 Da (+1.06 mmu/+1.62 ppm), MH+: 1955.84488 Da, RT: 22.31 min,

Identified with: SEQUEST (v1.20); XCorr:3.16, Probability:0.00, Ions matched by search engine: 27/88

Fragment match tolerance used for search: 0.6 Da

Fragments used for search: b; b-H_2O; b-NH_3; y; y-H_2O; y-NH_3

Protein references (1):

- Cystic fibrosis transmembrane conductance regulator OS=Mus musculus GN=Cftr PE=1 SV=2 - [CFTR_MOUSE]

MS/MS spectrum of monophosphorylated peptide TQITALKEETEEEVQETR phosphorylation site: T1467

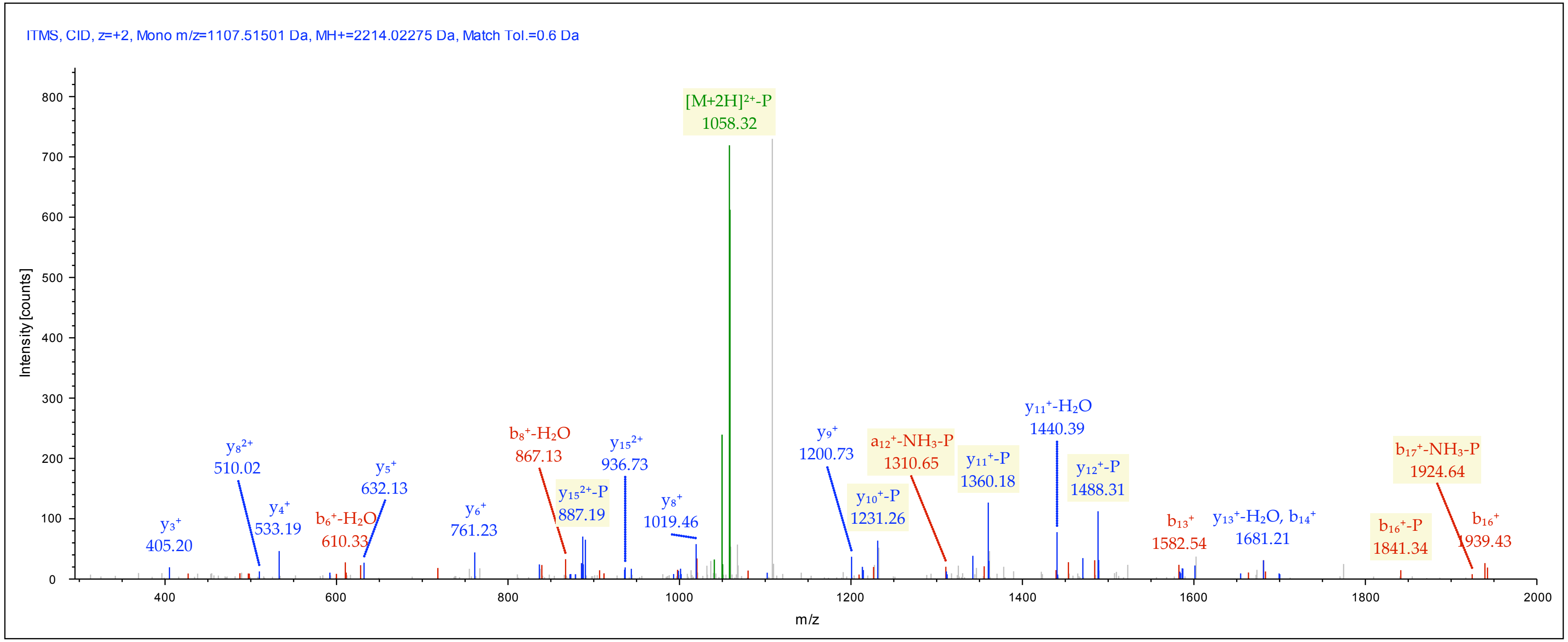

Sequence: TQITALKEETEEEVQETR, T10-Phospho (79.96633 Da)
Charge: +2, Monoisotopic m/z: 1107.51501 Da (+2.67 mmu/+2.41 ppm), MH+: 2214.02275 Da, RT: 21.48 min,
Identified with: Mascot (v1.27); IonScore:55, Exp Value:8.7E-004, Ions matched by search engine: 11/200
Fragment match tolerance used for search: 0.6 Da
Fragments used for search: a; a-H_2O; a-NH_3; b; b-H_2O; b-NH_3; y; y-H_2O; y-NH_3

Protein references (1):
- Cystic fibrosis transmembrane conductance regulator OS=Mus musculus GN=Cftr PE=1 SV=2 - [CFTR_MOUSE]

MS/MS spectrum of monophosphorylated peptide TQITALKEETEEEVQETR phosphorylation site: T1467

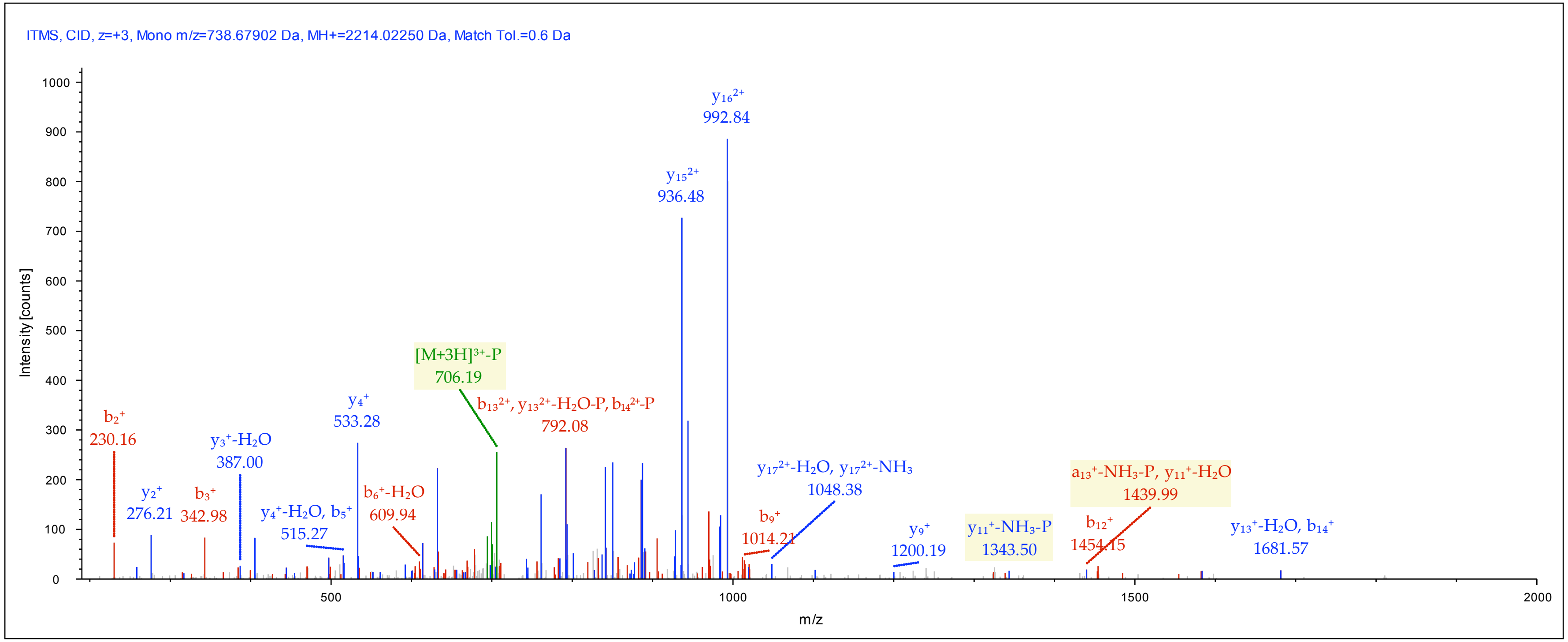

Sequence: TQITALKEETEEEVQETR, T10-Phospho (79.96633 Da)
Charge: +3, Monoisotopic m/z: 738.67902 Da (+1.69 mmu/+2.29 ppm), MH+: 2214.02250 Da, RT: 21.45 min,
Identified with: SEQUEST (v1.20); XCorr:5.05, Probability:0.00, Ions matched by search engine: 36/100
Fragment match tolerance used for search: 0.6 Da
Fragments used for search: b; b-H_2O; b-NH_3; y; y-H_2O; y-NH_3

Protein references (1):
- Cystic fibrosis transmembrane conductance regulator OS=Mus musculus GN=Cftr PE=1 SV=2 - [CFTR_MOUSE]
