# Supplementary material for: Detection of Phospho-Sites Generated by Protein Kinase CK2 in CFTR: Mechanistic Aspects of Thr1471 Phosphorylation
Source: PLoS One. 2013 Sep 18;8(9):e74232. doi: 10.1371/journal.pone.0074232 (PMC3776838; doi:10.1371/journal.pone.0074232)
Supplement: Table S1 — Phosphopeptides identified from mouse CFTR. For each peptide the following details are reported: aminoacid sequence, modifications, phosphorylation site/s, pRS score, pRS probability and pRS site probability, q-value, PEP score and number of missed-cleavages. (DOC) [file pone.0074232.s005.doc]

Table S1: Phosphopeptides identified from mouse CFTR.

| Sequence | Modifications | Phospo-site | pRS Score | pRS Probability | pRS Site Probabilities | q-Value | PEP | # Missed Cleavages |
| --- | --- | --- | --- | --- | --- | --- | --- | --- |
| RL**s**QDSTLNITEEINEEDLK | S3(Phospho) | S808 | 117 | 1,0 | S(3): 99.9; S(6): 0.1; T(7): 0.1; T(11): 0.0 | 0 | 0,000672994 | 1 |
| TQITALKEE**t**EEEVQETR | T10(Phospho) | T1467 | 108 | 1,0 | T(1): 0.0; T(4): 0.0; T(10): 100.0; T(17): 0.0 | 0 | 0,000571204 | 1 |
| RQ**s**VLDLMTFTPNSGSSNLQR | S3(Phospho) | S763 | 94 | 1,0 | S(3): 100.0; T(9): 0.0; T(11): 0.0; S(14): 0.0; S(16): 0.0; S(17): 0.0 | 0 | 0,1209925 | 1 |
| L**s**QDSTLNITEEINEEDLK | S2(Phospho) | S808 | 67 | 0,9 | S(2): 90.6; S(5): 4.7; T(6): 4.7; T(10): 0.0 | 0 | 0,008052224 | 0 |
| KN**s**ILNSFSSVR | S3(Phospho) | S698 | 94 | 1,0 | S(3): 100.0; S(7): 0.0; S(9): 0.0; S(10): 0.0 | 0 | 5,3466E-05 | 1 |
| LSLVPD**s**EQGEAALPR | S7(Phospho) | S737 | 95 | 1,0 | S(2): 0.0; S(7): 100.0 | 0 | 0,000431459 | 0 |
| RL**s**LVPDSEQGEAALPR | S3(Phospho) | S732 | 145 | 1,0 | S(3): 100.0; S(8): 0.0 | 0 | 0,05853575 | 1 |
| RRQ**s**VLDLMTFTPNSGSSNLQR | S4(Phospho) | S763 | 77 | 1,0 | S(4): 100.0; T(10): 0.0; T(12): 0.0; S(15): 0.0; S(17): 0.0; S(18): 0.0 | 0 | 4,92178E-06 | 2 |
| RL**s**QD**s**TLNITEEINEEDLK | S3(Phospho); S6(Phospho) | S808+S811 | 78 | 0,9 | S(3): 100.0; S(6): 93.4; T(7): 6.6; T(11): 0.1 | 0 | 0,002056111 | 1 |
| KI**s**IVQK | S3(Phospho) | S710 | 78 | 1,0 | S(3): 100.0 | 0 | 0,07584584 | 1 |
| RQ**s**VLDLMTFTPNSGSSNLQR | S3(Phospho); M8(Oxidation) | S763 | 100 | 1,0 | S(3): 99.8; T(9): 0.2; T(11): 0.0; S(14): 0.0; S(16): 0.0; S(17): 0.0 | 0 | 1,69782E-06 | 1 |
| RKN**s**ILNSFSSVR | S4(Phospho) | S698 | 51 | 1,0 | S(4): 99.7; S(8): 0.3; S(10): 0.0; S(11): 0.0 | 0 | 0,005446854 | 2 |
| EG**ss**DVLVIKNEHVK | S3(Phospho) | S1183 and/or S1184 | 0 | 0,5 | S(3): 50.0; S(4): 50.0 | 0 | 0,006704973 | 1 |
| KI**s**LVPQISLNEVDVYSR | S3(Phospho) | S790 | 79 | 1,0 | S(3): 100.0; S(9): 0.0; Y(16): 0.0; S(17): 0.0 | 0 | 0,08009747 | 1 |
| HHLELSDIYQAPSAD**s**ADHLsEK | S16(Phospho) | S45 and/or S50 | 23 | 0,7 | S(6): 0.1; Y(9): 0.2; S(13): 6.9; S(16): 21.0; S(21): 71.8 | 0 | 0,001367253 | 0 |
| RRQ**s**VLDLMTFTPNSGSSNLQR | S4(Phospho); M9(Oxidation) | S763 | 47 | 1,0 | S(4): 99.9; T(10): 0.0; T(12): 0.0; S(15): 0.0; S(17): 0.0; S(18): 0.0 | 0 | 0,03769125 | 2 |
| RF**s**VDDSSAPWSKPK | S3(Phospho) | S670 | 76 | 1,0 | S(3): 99.8; S(7): 0.1; S(8): 0.1; S(12): 0.0 | 0 | 2,66984E-07 | 1 |
| TPLCIDGE**s**DDLQEK | C4(Carbamidomethyl); S9(Phospho) | S723 | 50 | 1,0 | T(1): 0.0; S(9): 100.0 | 0 | 0,02412659 | 0 |
| SIFQQAIS**ss**EK | S10(Phospho) | S1439 and/or S1440 | 38 | 0,9 | S(1): 0.0; S(8): 11.0; S(9): 77.9; S(10): 11.0 | 0,005171 | 0,0288378 | 0 |
| EG**s**SDVLVIK | S3(Phospho) | S1183 | 59 | 0,9 | S(3): 92.2; S(4): 7.8 | 0,041249 | 0,1700099 | 0 |
| **ss**ILTE**t**LR | S2(Phospho) | S659 and/or S660 and/or T665 | 18 | 0,9 | S(1): 49.2; S(2): 49.2; T(5): 0.8; T(7): 0.8 | 0,041735 | 0,09673647 | 0 |

For each peptide the following details are reported: aminoacid sequence, modifications, phosphorylation site/s, pRS score, pRS probability and pRS site probability, q-value, PEP score and number of missed-cleavages.
