# Supplementary material for: Detection of Phospho-Sites Generated by Protein Kinase CK2 in CFTR: Mechanistic Aspects of Thr1471 Phosphorylation
Source: PLoS One. 2013 Sep 18;8(9):e74232. doi: 10.1371/journal.pone.0074232 (PMC3776838; doi:10.1371/journal.pone.0074232)
Supplement: Material S1 — (DOC) [file pone.0074232.s006.doc]

**Detection of phospho-sites generated by protein kinase CK2 in CFTR: Mechanistic aspects of Thr1471 phosphorylation.**

Andrea Venerando^1^, Cinzia Franchin^1,2^, Natasha Cant^3^, Giorgio Cozza^1^, Mario A. Pagano^4^, Kendra Tosoni^5^, Ateeq Al-Zahrani ^3,#^, Giorgio Arrigoni^1,2^, Robert C. Ford^3^, Anil Mehta^5^, Lorenzo A. Pinna^1*^

^1^Department of Biomedical Sciences, University of Padova, and CNR Institute of Neurosciences, Padova, Italy; ^2^Proteomic Center of Padova University, VIMM, and Padova University Hospital, Padova, Italy; ^3^Faculty of Life Sciences, University of Manchester, Manchester, UK; ^4^Department of Molecular Medicine, University of Padova, Padova, Italy; ^5^Division of Cardiovascular and Diabetes Medicine, Ninewells Hospital and Medical School, University of Dundee, Dundee, UK

**Supplementary Material**

**Expression and purification of the CFTR C-terminal 42-mer peptide.**

A pET-24a vector encoding the C-terminus of human CFTR was expressed in *E. coli* BL21 (DE3) cells as a 6 Histidine N-terminal fusion. Cells were grown at 37 °C in LB broth and when the OD600 reached 0.6 – 0.8 IPTG was added (1mM). The induced cultures were grown for 4 hrs at 25 °C and cells were centrifuged (10000 rpm , F10BCI-6x500Y Rotor, Beckman, 4 °C , 15 minutes). The pellets were stored at –20 °C, then were resuspended in 500 mM NaCl, 50 mM HEPES pH 7.0, 0.01% sodium azide (NaN_3_), 1 mM 2-mercaptoethanol, 0.1 % tritonX-100, 100 mg/ml lysozyme, 20 μg/mL PMSF (phenyl methane sulphonyl fluoride) before sonication for 5 s intervals followed by 10 s cooling period (10-15 cycles) at 30 % amplitude (Sonics Vibra Cell). The sample was centrifuged at 16000 rpm (JA-17 Rotor, Beckman, 20 min, 4 °C) and then protamine sulfate (1 mg/mL ) was added to the supernatant which was then centrifuged as above for 20 minutes. The pH was re-checked and adjusted to pH 7 if necessary.

Talon immobilized metal affinity chromatography (IMAC) resin was equilibrated with 500 mM NaCl, 50 mM HEPES pH 7.0, 0.01% sodium azide (NaN_3_), 1 mM 2-mercaptoethanol, 50 mM imidazol before mixing with the final supernatant (0.5 mL resin/2 l starting cell culture) in a Falcon tube and incubated at 4 °C for 1 hr. The contents were loaded into a 20 mL gravity flow column (Bio-Rad), and then washed with 10 column volumes of the above buffer. Elution was with 250 mM imidazole in the above buffer. The eluted protein was dialysed at 4°C overnight against 100 mM NaCl, 50 mM HEPES pH 7.9, 0.01% sodium azide (NaN_3_), 1 mM 2-mercaptoethanol,2mM CaCl_2_ using a 5000 molecular weight cut-off membrane (BDH Chemicals Ltd). Thrombin (Sigma, 200 units) and 2mM CaCl2 was added to the protein sample before it was placed into the dialysis membrane. A fresh batch of Talon resin was equilibrated using dialysis buffer before dialysed protein was added and the mixture was incubated at 4 °C for 1 hr before being loaded into a column and processed as above. The unbound material was collected and β-mercaptoethanol (10mM) and 2mM EDTA (ethylenediaminetetraacetic acid) were added. The protein was further purified and concentrated using Centricon centrifugal filter units (Millipore) by collecting the filtrate passing through a 50kDa cut-off filter, and then concentrating this with a 3 kDa cut-off filter. The purified polypeptide was concentrated to about 1mg/ml and stored at -80 °C.
